# Microbial drivers of DMSO reduction and DMS-dependent methanogenesis in saltmarsh sediments

**DOI:** 10.1038/s41396-023-01539-1

**Published:** 2023-10-25

**Authors:** Dennis Alexander Tebbe, Charlotte Gruender, Leon Dlugosch, Kertu Lõhmus, Sönke Rolfes, Martin Könneke, Yin Chen, Bert Engelen, Hendrik Schäfer

**Affiliations:** 1 Institute for Chemistry and Biology of the Marine Environment, University of Oldenburg, Carl-von-Ossietzky-Str. 9-11, 26129 Oldenburg, Germany; 2 School of Life Sciences, University of Warwick, CV4 7AL Coventry, UK; 3 Institute of Biology and Environmental Sciences, University of Oldenburg, Carl-von-Ossietzky-Str. 9-11, 26129 Oldenburg, Germany

**Keywords:** Microbial ecology, Soil microbiology

## Abstract

Saltmarshes are highly productive environments, exhibiting high abundances of organosulfur compounds. Dimethylsulfoniopropionate (DMSP) is produced in large quantities by algae, plants, and bacteria and is a potential precursor for dimethylsulfoxide (DMSO) and dimethylsulfide (DMS). DMSO serves as electron acceptor for anaerobic respiration leading to DMS formation, which is either emitted or can be degraded by methylotrophic prokaryotes. Major products of these reactions are trace gases with positive (CO_2_, CH_4_) or negative (DMS) radiative forcing with contrasting effects on the global climate. Here, we investigated organic sulfur cycling in saltmarsh sediments and followed DMSO reduction in anoxic batch experiments. Compared to previous measurements from marine waters, DMSO concentrations in the saltmarsh sediments were up to ~300 fold higher. In batch experiments, DMSO was reduced to DMS and subsequently consumed with concomitant CH_4_ production. Changes in prokaryotic communities and DMSO reductase gene counts indicated a dominance of organisms containing the Dms-type DMSO reductases (e.g., *Desulfobulbales*, *Enterobacterales*). In contrast, when sulfate reduction was inhibited by molybdate, Tor-type DMSO reductases (e.g., *Rhodobacterales*) increased. *Vibrionales* increased in relative abundance in both treatments, and metagenome assembled genomes (MAGs) affiliated to *Vibrio* had all genes encoding the subunits of DMSO reductases. Molar conversion ratios of <1.3 CH_4_ per added DMSO were accompanied by a predominance of the methylotrophic methanogens *Methanosarcinales*. Enrichment of *mtsDH* genes, encoding for DMS methyl transferases in metagenomes of batch incubations indicate their role in DMS-dependent methanogenesis. MAGs affiliated to *Methanolobus* carried the complete set of genes encoding for the enzymes in methylotrophic methanogenesis.

## Introduction

Saltmarshes are highly productive ecosystems important for coastal protection, biogeochemical cycling, and human nutrition [1, 2]. They are often characterized by distinct zones based on inundation frequencies and plant communities, affecting the composition of their nutrients and microbial communities [3–6]. Most of the biomass produced in saltmarshes is remineralized in the sediments [7]. While the majority of organic matter degradation in marine sediments (~50%) is driven by dissimilatory sulfate reduction [8], other energetically more favorable electron acceptors, such as dimethyl sulfoxide (DMSO), may also play a key role [9, 10].

**Fig. 1:**
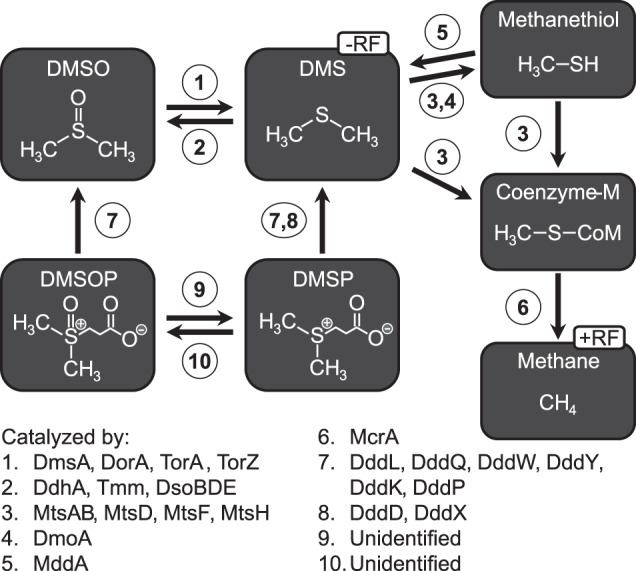
Schematic summary of relevant organosulfur compounds and enzymes. DMS and CH_4_ have contrasting negative and positive effects on radiative forcing (−RF, +RF). DMSO is reduced by DMSO reductases (DmsA, DorA, TorA, TorZ) and can be oxidized by DMS dehydrogenase (DdhA), trimethylamine (TMA)-monooxygenase (Tmm) or by a multicomponent monooxygenase (DsoBDE). DMS can be oxidized to methanethiol (MeSH) by DMS monooxygenase (DmoA). DMS methyltransferases (MtsAB, MtsD, MtsF, MtsH) catalyze the methyl transfer from DMS with methanethiol (MeSH) as an intermediate. The produced methyl-coenzyme M is reduced by the methyl-coenzyme M reductase (McrA) under CH_4_ formation. The degradation of DMSP is mediated by a wide range of DMSP lyases (DddL, DddQ, DddW, DddY, DddK, DddP, DddD, DddX), of which most can also degrade DMSOP. The enzymes involved in the conversion between DMSP and DMSOP remain unidentified.

Respiratory reduction of DMSO under anoxic conditions yields dimethylsulfide (DMS) [11], a trace gas that acts as a precursor for secondary organic aerosols, which may have a climate-cooling effect [12–14]. A precursor for DMS and DMSO is dimethylsulfoniopropionate (DMSP), produced by plants, algae, and bacteria [15–18]. Thus, the high prevalence of these DMSP-producing organisms in saltmarshes fosters organic sulfur cycling (Fig. 1). Previous work demonstrated that DMSO addition to anaerobic saltmarsh sediment slurries led to production of DMS, which in turn was consumed with concomitant production of CH_4_ [19, 20]. The cycling of DMSO in saltmarsh sediments thus affects the production of compounds with positive (CO_2_ and CH_4_) and negative radiative forcing (DMS). For their small global surface area of ~0.01% [21], saltmarshes contribute an overproportioned amount (~0.28%) to global DMS [22] and marine CH_4_ emissions (~13%) [23]. Understanding the fates of DMS and DMSO in these carbon sequestering (blue carbon) ecosystems is therefore important for modeling their effect on climate, as well as carbon and sulfur turnover [24].

Sources of DMSO in the marine realm include bacterial production, photochemical oxidation of DMS in seawater, and atmospheric precipitation [13, 25]. Bacterial DMSO production is linked to enzymatic activities of a DMS dehydrogenase (catalytic subunit DdhA), as demonstrated for *Rhodovulum sulfidophilum* [10, 26]. Furthermore, co-oxidative turnover of DMS by trimethylamine monooxygenase (Tmm) was found in representatives of highly abundant marine microorganisms (e.g., *Pelagibacter ubique* (SAR11) and *Ruegeria pomeroyi*) [27, 28]. *Acinetobacter* sp. strain 20B was found to use a multicomponent monooxygenase (DsoBDE) for DMS oxidation to DMSO [29]. DMSO is also produced during degradation of dimethylsulfoxonium propionate (DMSOP) found in bacteria and algae [30], and six DMSP lyases were demonstrated to catalyze the cleavage of DMSOP [31]. DMSO production has also been shown in certain *Rhodobacterales* [32], *Flavobacteriales* [33], and, more recently, in diatoms [34], but the enzymatic basis remains unidentified in these organisms.

Microbial DMSO reduction in anoxic saltmarsh sediments has not been characterized beyond process measurements in slurry incubations [19, 20]. The microorganisms driving DMSO reduction and the cycling of DMS under anaerobic conditions in these environments remain poorly characterized. Indeed, anaerobic DMSO reduction- and DMS degradation experiments showed that different microbial community members of saltmarsh sediments compete for these compounds [19, 35, 36]. In these experiments, their metabolic conversions led to the production of CH_4_ or CO_2_, with DMS and methanethiol (MeSH) as intermediates. The fate of DMS may depend on substrate concentrations, with methanogenic archaea dominating at high DMS concentrations and sulfate-reducing bacteria (SRB) at low concentrations [19, 37]. The reduction of DMSO is catalyzed by DMSO reductases (DMSOR), of which two major types have been described in *Escherichia coli* and *Rhodobacter* species, referred to as the Dms- and Dor-types, respectively [38, 39]. These belong to the larger family of DMSO-reductase family enzymes [10], also including closely related trimethylamine-*N*-oxide reductases, which are often bifunctional, and which can (TorZ) or may (TorA) also reduce DMSO [9, 40].

While the enzymes driving anaerobic DMS degradation in bacteria remain unidentified, those catalyzing methanogenic DMS degradation have been characterized in *Methanosarcina* species. For instance, in *Methanosarcina barkeri*, a methyltransferase complex composed of the subunits MtsAB transfers the methyl groups from DMS to coenzyme-M forming methyl-CoM, a key component in methanogenesis [41]. *Methanosarcina acetivorans*, in turn, can use the two-domain proteins MtsD or MtsF, which carry out a two-step methyl transfer under methyl-CoM formation [42, 43]. Although putative DMS-dependent methanogenic populations were recently described for marine and intertidal sediments [36, 44], their identity in saltmarsh sediments remains unknown.

Our investigation aimed to close the knowledge gaps in identifying and quantifying compounds, organisms, and key genes involved in DMSO reduction and DMS degradation within characteristic saltmarsh zones. We hypothesize that: (i) the concentrations of organic sulfur compounds differ between the investigated zones, (ii) DMS from DMSO reduction can further be degraded by specific methanogens, and (iii) genes encoding for the enzymes involved in these processes are overrepresented in corresponding metagenomes from batch experiments and environmental samples. To test these hypotheses, we measured DMSO and DMSP in two different saltmarshes (located on Spiekeroog Island, Germany, and Stiffkey, Norfolk, UK, Fig. S1, Tab. S1). These were sampled across defined zones and various sediment depths. Samples from selected sites were subjected to anaerobic batch incubation experiments to follow the succession of DMSO reduction and DMS degradation. Microorganisms driving DMSO reduction and DMS degradation were identified based on statistical analysis of changes in ribosomal RNA gene diversity and determination of genes encoding for DMSOR and DMS methyltransferases in metagenomes from incubations and natural saltmarsh communities.

## Materials and methods

### Sampling

Sediments were sampled from the back barrier reef of the North Sea Island Spiekeroog, Germany (July 2019, September 2019, March 2020) and the Stiffkey saltmarsh, United Kingdom (July 2021, Fig. S1). All samples were taken as push cores using sterile cut-off syringes to obtain the corresponding volumes (e.g., 1 cm^3^) and weighed for density (Fig. S2, Tab. S1). Sediments from Spiekeroog (0–1 cm, 4–5 cm) were sampled along three land-to-sea transects in the upper saltmarsh (Upp), lower saltmarsh (Low) and pioneer zone (Pio), with additional sites from the shoreline (Edge) and the intertidal mudflat (Mud). Depth profiles were sampled in 1 cm steps (0–6 cm) from Spiekeroog (Pio, Edge) and Stiffkey: *Spartina-*vegetated sites (Spar), bare areas with sandy sediments (Sand), and muddy pools (Pool). Additionally, bulk sediments combining the top 5 cm were sampled at Pio, Edge, and Pool for incubation experiments and kept at 4 °C until further use. All samples for molecular analyses and DMSO measurements were frozen on site, samples for DMSP quantification were supplemented with 5 ml 5 M NaOH and sealed in the field. Transport and further processing for chemical analysis was carried out in sterile glass tubes, crimp-sealed with new butyl rubber stoppers.

### Headspace gas chromatography

DMSP and DMSO concentrations were measured in duplicates as DMS by headspace gas chromatography after chemical DMSP lysis [45] or DMSO reduction [46]. While the sample preparation in the field already induced chemical lysis of DMSP, chemical DMSO reduction was done by suspending 1 cm^3^ sediment in 5 ml ddH_2_O, 18 min nitrogen stripping [47], and then supplemented with 3.85 g Na_2_S_2_O_5_ as reductant prior to incubation at 60 °C for 10 min, 50 °C for 30 min, and 1 °C for 5 min. Headspace gas chromatography of DMS and CH_4_ was done at 30 °C sample temperature. Samples from Spiekeroog were measured on an SRI 8610 C gas chromatograph (SRI Instruments, Bad Honnef, Germany) with a Siltek-treated stainless-steel column (No. 70139–273; 30 m by 0.28 mm, MXT-1 coated; Restek, Bad Homburg, Germany), with an oven temperature of 51 °C, argon as mobile phase, and a flame ionization detector (FID, 382 °C). Peak integrations were done with Peak Simple V3.56 by SRI Instruments. Samples from Stiffkey were measured on a Shimadzu GC2010plus as described previously [28]. DMS, DMSO, and DMSP standards were treated and measured as samples. The gas mixture N6 (No. 795.05106; Air Liquide, Düsseldorf, Germany) was used for CH_4_ standards. Concentrations were calculated assuming complete degassing of CH_4_ to the head space.

### Incubation experiments

Material from three cores per site (Pio, Edge, Pool) was combined and homogenized (Fig. S1, Tab. S1). Approximately 2 cm^3^ sediment was transferred to 100 ml serum bottles, with 20 ml sterile anoxic basal medium. The medium was adapted from elsewhere [48] and contained per liter of ddH_2_O: 0.2 g KH_2_PO_4_, 0.25 g NH_4_C1, 20 g NaC1, 3 g MgC1_2_ ∙ 6H_2_O, 0.15 g CaC1_2_ ∙ 2H_2_O, 0.3 g KC1, 1.5 g Na_2_SO_4_, and 0.25 mg resazurin as oxygen indicator. The following components were added from sterile stock solutions to the autoclaved media: 1 ml trace element solution SL10 [49], 1 ml selenite-tungstate solution, 30 ml 1 M NaHCO_3_, 1 ml 0.2 M Na_2_S [48], and 2 ml 7-vitamin [50] or 10 ml 10-vitamin solution [51], for Spiekeroog and Stiffkey experiments, respectively. All transfers were done while flushing with N_2_-gas to keep the media anoxic after oxygen removal by autoclaving. Anoxic sediment incubations from Spiekeroog in July 2019 (Pio, Edge) and Stiffkey in July 2021 (Pool) were set up in triplicates, supplemented with 1 mM DMSO (DMSO), 1 mM DMSO + 10 mM sodium molybdate (DMSO + Mo), or without any addition (Control). The sulfate reduction inhibitor molybdate (Na_2_MoO_4_) was added to assess the potential role of sulfate reducers for DMSO reduction and DMS removal. The start (T0) and endpoints (T2) were sampled for molecular analysis. Depletion of DMS and plateau CH_4_ concentrations defined the endpoints. Due to the destructive sampling, an additional triplicate was set up for the Stiffkey experiments to target peak DMS concentrations (T1). Additional experiments with samples from Spiekeroog (Sep 2019) were performed to compare the effect of different DMSO concentrations on DMS and CH_4_ yields at 1 mM DMSO (DMSO) to 0.1 mM DMSO (0.1 mM DMSO). DMS and CH_4_ production were monitored as described above throughout all experiments.

### DNA extraction and sequencing

For each saltmarsh site, triplicates of 1 cm^3^ were mixed to create bulk sediment samples. The sediment fractions of the incubation experiments were collected by centrifugation, discarding the supernatants. DNA extractions were performed with 0.25–0.5 g sediment using the DNeasy PowerSoil Pro Kit (Qiagen, Hilden, Germany) according to the manufacturer’s instructions. DNA quality and concentrations were controlled with a NanoDrop 2000c (Thermo Fisher Scientific, Bremen, Germany), and samples were stored at −20 °C until further use. 16S rRNA gene sequencing of the V4-V5 region was done with the 515F-Y (5′-GTGYCAGCMGCCGCGGTAA-3′) and 926 R (5′-CCGYCAATTYMTTTRAGTTT-3′) primers [52]. Amplification and library preparation was performed as described previously [53] on a NovaSeq PE250 platform (Illumina, Berlin, Germany). Shotgun metagenome sequencing was done by paired-end sequencing (2 × 150 bp) with a NovaSeq 6000 system (Illumina) using samples from the Spiekeroog sites (July 2019) and Stiffkey incubations.

### Processing of 16S rRNA gene amplicon data

16S rRNA gene sequence data were processed to amplicon sequencing variant (ASV) counts with qiime2-2021.2 [54] as a wrapper for the denoising algorithm DADA2, as described previously [55]. The scripts were adapted from the collection available at: https://github.com/jcmcnch/eASV-pipeline-for-515Y-926R and used in a standardized conda environment. Primer- and general trimming were done with cutadapt [56], allowing a primer sequence mismatch of 20%. Subsequently, 16S rRNA gene sequences were separated using bbtools and SILVA138 [57]. Low-quality ends (median quality score <30) were removed by trimming the forward and reverse sequences at a sequence length of 220 bp. Subsequent steps for denoising, merging, and chimeric removal were done with qiime2 dada2 [58]. Taxonomy was assigned using the qiime2 classify-sklearn plugin and SILVA138 as reference database. Sequences assigned to chloroplasts or mitochondria were excluded.

### Metagenome assembly and sequence classification

Metagenomes were assembled as described elsewhere [59] with the following modifications: Metagenomes from Stiffkey incubations, gene sequences were clustered at 95% identity using usearch 10.0.24 [60] (*-cluster_fast –id 0.95)* to generate a non-redundant gene catalog as described before, resulting in 8.51 × 10^6^ representative gene sequences and 1.62 × 10^9^ mapped reads (65% of total sequences). Due to the high sequence diversity and deep sequencing of the Spiekeroog metagenomes, clustering was not possible. Thus, duplicate sequences were removed from the gene catalog using usearch 10.0.24 [60] (*-fastx_uniques*), resulting in 4.44 × 10^7^unique sequences and 1.64 × 10^9^ mapped reads (75% of total sequences). Sequences were taxonomically (Kaiju 1.6) [61] and functionally (GhostKOALA) [62] classified as described before. Different sequencing depths and gene lengths were accounted for by dividing read counts by the gene length in kb forming reads per kilobase (rpk) and subsequently dividing their sums per sample by one million (rpkm). The methylthiol:coenzyme M methyltransferase MtsAB and methyl transferases MtsDFH (homologs of loci MA0859, MA4384, MA4558 of *Methanosarcina acetivorans*) [42] were identified with DIAMOND BLASTp [63] with Q48924, Q8PUA8, and Q48925 (UniProtKB/Swiss-Prot), as well as WP_048064984.1, WP_011024263.1, and WP_011024431.1 (NCBI) as references (>70% amino acid identity, *e*-value < 1 × 10^−29^).

### Metagenome binning and functional classification

Contig coverage was determined using SAMtools v1.15.1–12g31dbb4 [64] and subsequently binned using metaBAT2 v2 [65]. The resulting bins were evaluated using CheckM v1.2.0 [66] and CheckM2 v1.0.2 [67] for bins from Spiekeroog and Stiffkey metagenomes, respectively. Only good quality bins (>50% completeness, <5% contamination) were included in further metagenome assembled genome (MAG) analyses. 5.80 × 10^8^ (23%) and 5.39 × 10^8^ (25%) reads mapped to the MAGs assembled from Stiffkey and Spiekeroog samples, respectively. MAGs were phylogenetically classified using the GTDB-Toolkit *classify_wf* [68]. Genes were functionally classified using GhostKOALA and DIAMOND BLASTp, as described above. Given the much higher mapping rates from the metagenomes (65%, 75%) compared to the MAGs (23%, 25%), most analyses were done utilizing the higher resolution of the metagenomes, while individual MAGs were analyzed to provide information on potential metabolic pathways.

### Statistical analyses and visualization

All statistical analyses and data visualizations were done with R version 4.1.2 [69], using tidyverse v 2.0.0 [70] for most data-wrangling tasks. Differentially abundant ASVs were identified with DESeq2 [71], comparing samples grouped by treatment, time point, and site. Therefore, 16S rRNA gene sequences were filtered to have ≥20 reads in ≥3 samples, and 1 read was added to the input matrix as deseq2 cannot handle 0 entrances. Shrunken log2FoldChanges (LFC) and SE [72] were added, and variance stabilizing transformation was applied. ASVs with Benjamini-Hochberg adjusted *p* values ≤ 0.05 and a log2FoldChange ≥ 0.5 were considered as significantly increased compared to controls.

## Results

### DMSP and DMSO concentrations increased towards the shoreline and decreased with sediment depth

**Fig. 2:**
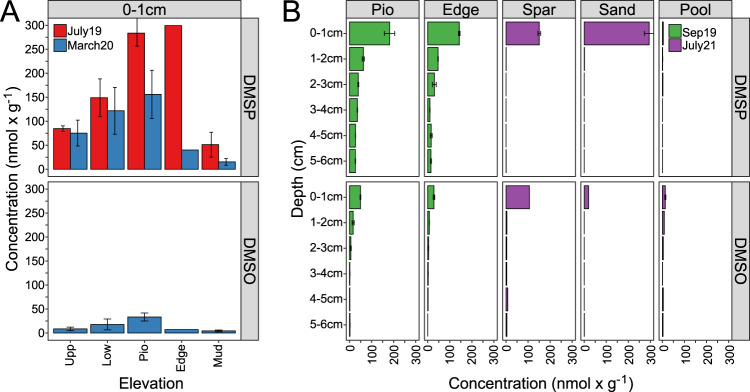
DMSP and DMSO profiles of saltmarsh sampling sites. **A** Land to sea transect on Spiekeroog (DE). **B** Depths profiles of DMSP and DMSO concentrations at selected sites from Spiekeroog (green) and Stiffkey saltmarsh (purple). Sampling campaigns were conducted in September 2019 (Spiekeroog) and July 2021 (Stiffkey).

While DMS was not detected in any sediment sample, DMSP and DMSO were found in most samples at Spiekeroog and Stiffkey (Fig. 2, Tab. S2). On Spiekeroog, DMSP and DMSO concentrations increased towards the pioneer zone (Pio, March 2020) and shoreline (Edge, July 2019). DMSP concentrations in July 2019 (154 ± 102 nmol∙g^−1^) exceeded those of samples taken in March 2020 (88 ± 63 nmol∙g^−1^). All depth profiles showed steep decreases within the upper 2 cm from 155 ± 97 (0–1 cm) to 23 ± 28 nmol∙g^−1^ (1–2 cm) for DMSP and 36 ± 11 (0–1 cm) to 9 ± 6 nmol∙g^−1^ (1–2 cm) for DMSO. When both compounds were measured simultaneously (Sep 2019, March 2020, July 2021), DMSP concentrations exceeded those of DMSO 3- to 4-fold (DMSP: 43 ± 69 nmol∙g^−1^, DMSO: 11 ± 17 nmol∙g^−1^). Exceptions were samples from the Stiffkey mud pool (Pool), where DMSO concentrations were higher than those of DMSP with 13.3 ± 2.3 nmol∙g^−1^ and 4.2 ± 0.1 nmol∙g^−1^ at 0−1 cm, respectively. However, the mud pool showed with 1.4 ± 1.4 and 5.7 ± 4.2 nmol∙g^−1^ generally the lowest mean DMSP and DMSO concentrations across the depth profiles. The high DMSO concentrations in the sediment samples (average 11 nmol∙g^−1^, maximum 106 nmol∙g^−1^) demonstrate the availability of DMSO as a respiratory electron acceptor in these saltmarsh sediments.

### Genes and organisms involved in organic sulfur cycling were identified by metagenome analysis

**Fig. 3:**
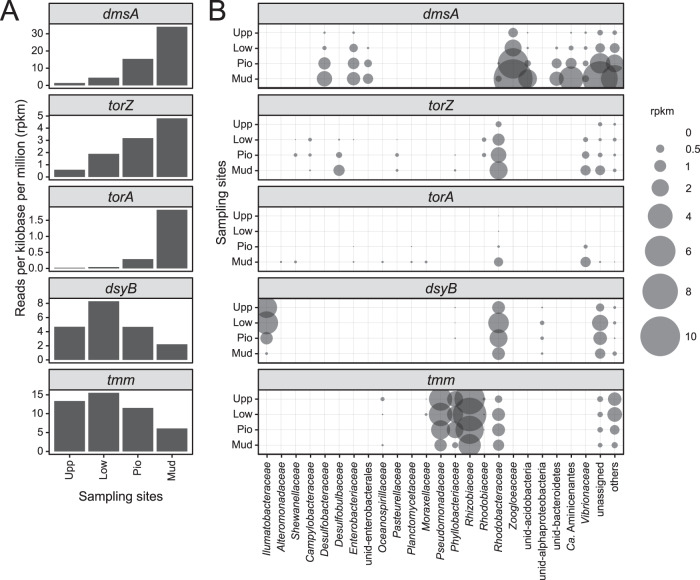
Gene counts and taxonomic affiliation of DMSO reduction genes across the land-to-sea transect at Spiekeroog. **A** Mean (*n* = 3) total counts per gene and zone (Upp, Low, Pio, Mud). **B** Mean (*n* = 3) total counts per gene and sample, with taxonomic affiliation. “Others” are groups with <2% of total counts of the respective gene.

Metagenome analysis of the Spiekeroog transects (Fig. 3) showed peak gene abundances for DMSP production (*dsyB*) and DMS co-oxidation to DMSO (*tmm*) in the lower saltmarsh (Low). The discrepancy of the peak bacterial DMSP and DMSO production genes to the peak concentrations of these compounds (Fig. 2) hints towards alternative sources. The genetic potential for DMSO respiration represented by the *torA*, *torZ*, and *dmsA* genes consistently increased from land to sea. This mostly matched the trend of DMSO concentrations, except for the high DMSOR gene counts accompanied by lower DMSO concentrations in the mudflat samples (Mud). The *dmsA* gene was abundant in *Zoogloeaceae, Enterobacterales*, and *Desulfobacteraceae* with up to 9.6 ± 1.0, 3.1 ± 0.3, and 1.6 ± 0.2 rpkm (all Mud), respectively. The Tor-type gene counts were generally lower compared to *dmsA*. Taxa with one of these DMSOR genes were for instance affiliated to *Rhodobacteraceae*, *Vibrionaceae*, *Pasteurellaceae*, *Desulfobulbaceae*, and *Campylobacteraceae*. Some of the above-mentioned taxa were also detected by 16S rRNA gene analysis (Fig. S3). For example, the *Rhodobacteraceae* were ubiquitous in all environmental samples and the SRB (e.g., *Desulfobacteria*, *Desulfobulbia*) increased in relative abundance towards the sea. All analyzed genes involved in DMSO reduction, as well as DMSP and DMSO production, were found within the *Rhodobacteraceae*, underlining their key role in organic sulfur cycling in saltmarshes. Phylogenetic analysis of DMSOR detected in MAGs and from the metagenome assembly ( > 600 amino acids) demonstrated that the vast majority of these consistently clustered with reference sequences of DmsA, TorA, and TorZ (Fig. S6). Only some sequences fell into separate clades without clear affiliation to these DMSOR proteins or to the closely related biotin sulfoxide reductase (BisC). With a few exceptions (sequences: bin 148-2, ID434, ID1597, ID235, and ID1351) all of the TorZ and TorA sequences used for phylogenetic tree analysis had export signals for the TAT protein export pathway (SignalP v5.0 [73]), whereas these were not present in the BisC enzymes of *E.coli* (P20099) and *Cereibacter sphaeroides* (P54934).

### DMSO addition in incubation experiments stimulates the enrichment of DMSO reducers detected in saltmarsh sediments

**Fig. 4:**
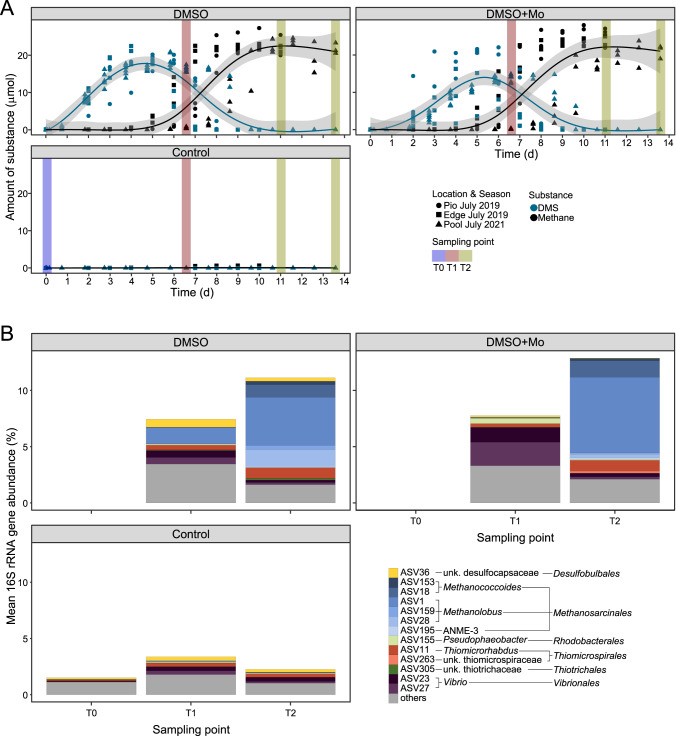
DMSO incubation experiments with saltmarsh sediment samples from Spiekeroog (Pio and Edge, July 2019) and Stiffkey (Pool, July 2021). **A** DMS (blue) and CH_4_ (black) production in incubation experiments. Sampling points for molecular analyses are marked with colored bars, with T0 at the beginning of the experiment (blue), T1 after peak DMS concentrations (red, only Stiffkey), and T2 (11d Spiekeroog, 13.5d Stiffkey) at the ends of the experiments designated by plateau CH_4_ concentrations and complete DMS removal (green). **B** Mean relative 16S rRNA gene abundances of all three experiments (Pio, Edge, Pool) of statistically significantly increased ASVs due to treatment (*n* = 3, deseq2, *p* ≤ 0.05, log2 fold change ≥ 0.5). Displayed ASVs had a relative abundance of >1% in at least one sample, while the remaining are grouped into “Others”.

All experiments amended with DMSO led to DMS production from the start of the incubation (Fig. 4A). They showed no detectable lag phase (Fig. S4), indicating the presence of an established community capable of DMSO reduction. DMS production from intrinsic sources of the 2 cm^3^ sediment in the controls was not detected, suggesting DMSO reduction as the sole origin of DMS. Considering that we used bulk sediment (0–5 cm) with varying environmental DMSO concentrations (see above) and 100 ml serum flasks (large headspace), the expected DMS yields in the controls were close to or below detection limits (~30 nmol in serum bottles). Stoichiometrically, a complete turnover of 1 mM DMSO within the 20 ml-batch incubations would yield a maximum of 20 µmol DMS. Peak DMS yields ranged from 18.4 ± 1.8 to 20.3 ± 2.5 µmol in DMSO treatments and from 13.2 ± 8.7 to 21.1 ± 0.5 µmol in DMSO+Mo, corresponding to molar conversion ratios of 0.7–1.1 DMS per added DMSO (Tab. S3).

While some shifts in relative 16S rRNA gene abundances were clearly visible at higher taxonomic levels (e.g., *Methanosarcinaceae*), minor changes (e.g., *Rhodobacteraceae*) were more difficult to depict in an overview bar chart (Fig. S5). For this, we used differential community analysis (DESeq2) of 16S rRNA gene counts on ASV level, revealing significant increases (*p*. ≤ 0.05, log2 fold change ≥ 0.5) of specific taxa in DMSO and DMSO+Mo treatments compared to the control (Fig. 4B). For instance, *Vibrionales* increased in relative abundance in DMSO and DMSO+Mo at peak DMS concentrations (T1). Furthermore, *Rhodobacterales* only increased in DMSO + Mo at T1, while SRBs (*Desulfobacterales* and *Desulfobulbales*) were enriched in DMSO treatments.

**Fig. 5:**
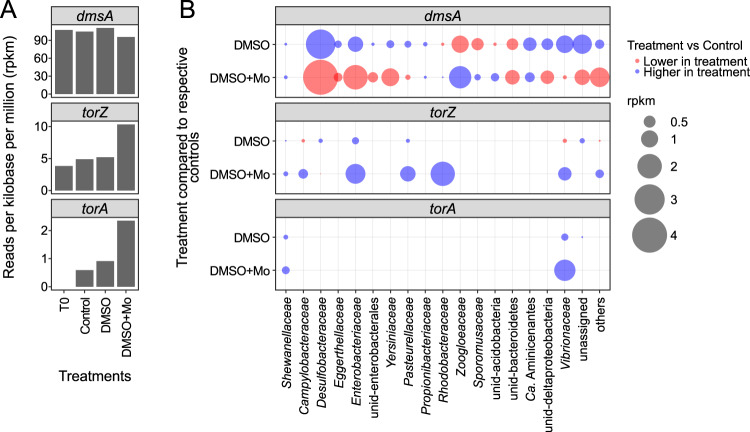
Gene counts and taxonomic affiliation of DMSO reduction genes in the incubations from Stiffkey saltmarsh at peak DMS concentrations (T1). **A** total counts per gene and sample. **B** treatment-dependent change in counts with taxonomic affiliation. The counts were derived by subtracting the Control from the treatments (DMSO, DMSO + Mo). Negative and positive values are lower- (red) or higher (blue) in treatment compared to the Control, respectively. “Others” are groups with <2% of total counts of the respective gene.

All mentioned taxa identified by differential analysis of 16S rRNA genes represent lineages for which DMSO reduction was experimentally proven (Tab. S4) or groups whose members were shown to contain one of the DMSO reductase genes *dmsA*, *torA*, and *torZ* within the metagenomes (Fig. 5). Similar to the environmental sites on Spiekeroog, *dmsA* genes had the highest numbers (105 ± 6 rpkm), *torZ* (6 ± 3 rpkm), and *torA* (1 ± 1 rpkm) in the metagenomes from Stiffkey incubations. Although the total counts of *dmsA* genes were similar between controls and treatments, differences were found in taxonomic affiliations. The *dmsA* gene reads affiliated to for example *Desulfobacteraceae*, *Enterobacteriaceae*, and *Vibrionales* increased in the DMSO treatment, but decreased in DMSO + Mo incubations. An opposite trend was observed in the *Zoogloeaceae* (all *Thauera sp*. MZ1T), which showed higher *dmsA* counts in DMSO + Mo treatments. Candidatus *Aminicenantes* was one of the few taxa which showed elevated *dmsA* counts in both treatments. While the overall numbers of the Tor-type of DMSOR (*torA*, *torZ*) was lower compared to *dmsA*, they substantially increased due to the treatments. This was observed in the genes affiliated to *Shewanellaceae*, *Enterobacteriaceae*, *Pasteurellaceae*, and *Vibrionaceae* in both treatments and for *Rhodobacteraceae* in DMSO + Mo only.

The above-mentioned bacteria which increased in the 16S rRNA gene datasets from the incubation experiments also carried DMSOR genes in the environmental samples from Spiekeroog. The analysis of the metagenomes led to 80 and 331 MAGs for environmental saltmarsh samples (Spiekeroog) and incubations (Stiffkey), respectively. In total, 14 MAGs contained either one of the investigated genes for DMS oxidation or DMSP production (Tab. S5). Only 3 MAGs, all affiliated to the Genus *Vibrio*, carried at least one of the DMSOR genes encoding for DmsBAD, TorAC, or TorZY. Furthermore, they also carried sequences encoding for the TorS, TorT, TorD, and TorR, while DmsC (K00185, K07308) could not be found.

### The majority of DMS produced by DMSO reduction is subject to DMS-dependent methanogenesis

DMSO reduction to DMS in the incubation experiments was followed by a decrease in DMS concentrations and simultaneous CH_4_ formation (Fig. 3A). The amounts of CH_4_ produced within the controls were neglectable ( ≤ 0.3 ± 0.3 µmol). In some experiments, CH_4_ production had started before the DMS peaks were reached, presumably reducing its peak concentrations and apparent molar conversion ratios. Taking a stoichiometric ratio of 1.5 mol CH_4_ per mol DMS in DMS-dependent methanogenesis into account [74], the maximum yield from the added 20 µmol DMSO would therefore be 30 µmol CH_4_. In our experiments, peak CH_4_ concentrations were between 21.0 ± 1.8 and 26.1 ± 2.4 µmol, corresponding to a yield of 1.1–1.3 CH_4_ per DMSO. Hence, most of the DMS from DMSO reduction was used for DMS-dependent methanogenesis. Slight differences were observed between DMSO+Mo and DMSO treatments for Edge (24.3 ± 1.4 vs. 22.2 ± 0.8 µmol) and Pio incubations (26.1 ± 2.4 vs. 23.6 ± 3.2 µmol), potentially due to the inhibition of DMS-degrading SRB and the subsequently higher availability for DMS dependent methanogenesis. However, this trend was reversed in the Pool incubations (21.0 ± 1.8 vs. 23.8 ± 0.8 µmol). The degradation of DMS to CH_4_ was expected to generate reduced inorganic sulfur compounds, such as hydrogen sulfide (H_2_S). The relative abundance of sulfur-oxidizing *Thiotrichales* and *Thiomicrospirales* increased in both treatments over time, reaching up to 2% ± 1 for *Thiomicrorhabdus* in DMSO+Mo treatments at T2.

In additional experiments (Sep 2019), DMS yields peaked in 0.1 mM DMSO treatments after 2 days of incubation (Fig. S3) and, therefore, earlier than under higher DMSO concentrations (6 days). With molar conversion ratios of 0.3 and 0.5 in Edge and Pio incubations, respectively, the CH_4_ yield per DMSO was lower in the 0.1 mM incubations compared to 0.4 and 1.1 in the corresponding Edge and Pio 1 mM DMSO treatments (Tab. S3). This suggests that DMSO concentration and resulting DMS production affected the competition for DMS between methanogens and alternative DMS degraders.

### MtsD and MtsH were identified as the dominant methyltransferases in DMS-dependent methanogenesis

DMS-dependent methanogenesis was accompanied by a significant increase in the relative abundance of *Methanosarcinales* in the 16S rRNA gene datasets (Fig. 4), accounting for up to 17% ± 14 of the total prokaryotic community (T2). This was mainly driven by *Methanolobus* spp. in the Stiffkey samples (40% ± 1.7), with other archaeal taxa only increasing slightly at T2 (*Methanococcoides*, ANME-3). Members of *Methanolobus* and *Methanococcoides* were previously reported to be methylotrophic methanogens [75]. They increased in relative abundance during the DMS consumption and CH_4_ production phase, suggesting they were the primary DMS consumers in the incubations.

**Fig. 6:**
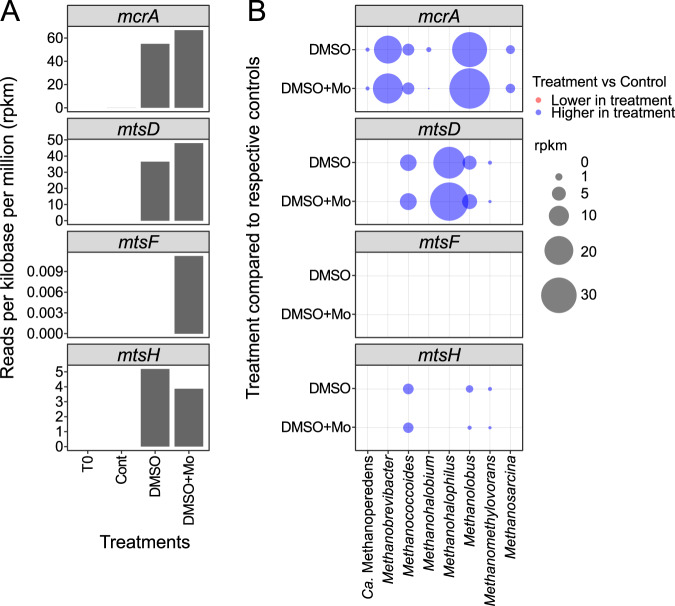
Gene counts and taxonomic affiliation of *mcrA* (K00399, K00400) and *mtsDFH* in the incubations from Stiffkey saltmarsh. **A** Total counts per gene and sample. **B** Treatment-dependent change in counts with taxonomic affiliation. The counts were derived from the subtraction of the respective control from the treatment (e.g., T1-Cont subtracted from T1-DMSO). Negative and positive values are lower- (red) or higher (blue) in treatment compared to the control, respectively.

We screened all metagenomes for the methyl-coenzyme M reductase (*mcrA*), the marker gene for methanogenesis, the methyltransferase, and corrinoid subunits of the methylthiol:coenzyme M methyltransferase (*mtsA, mtsB*) (Fig. 6). While *mcrA* genes were enriched in the incubations (T2) and were found at very low numbers (<0.2 rpkm) in the Spiekeroog transect, homologs of *mtsA* and *mtsB* were not found in any sample. However, we performed an additional DIAMOND BLASTp search including *mtsAB* and the fused corrinoid/methyltransferase proteins MtsDFH, implicated in DMS-dependent growth in *Methanosarcina acetivorans*. While MtsAB were also not found by this method, MtsDFH homologs affiliated with *Methanosarcinaceae* could be retrieved from incubation experiment metagenomes (DMSO, DMSO + Mo). All identified MtsDFH homologs were previously unassigned by GhostKOALA.

**Fig. 7:**
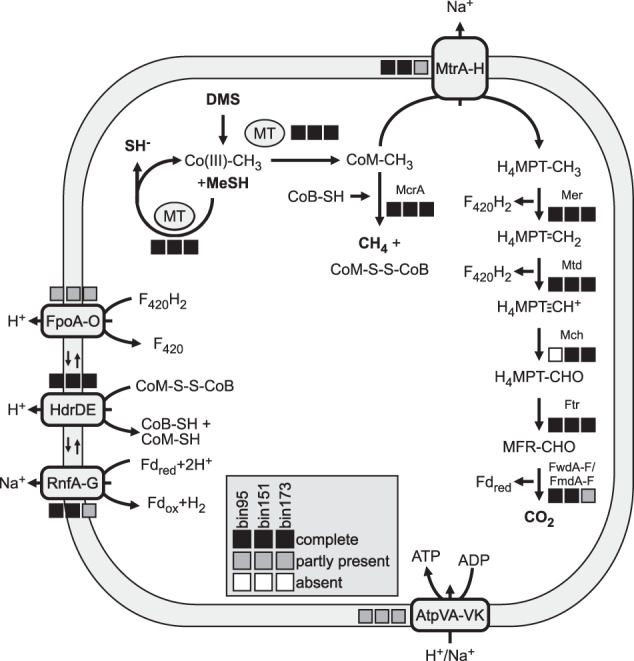
Schematic overview of the potential pathway used for DMS-dependent methanogenesis in *Methanolobus*. The boxes indicate the presence of all (black), some (gray) or absence (white) of the genes encoding specified enzymes in the three MAGs of *Methanolobus* (bin95, bin151, bin173). MT methyltransferase, McrA methylcoenzymeM reductase, MtrA-H tetrahydromethanopterin S-methyl-transferase, Mer 5,10-methylenetetrahydromethanopterin reductase, Mtd methylenetetrahydromethanopterin dehydrogenase, Mch methenyltetrahydromethanopterin cyclohydrolase, Ftr formylmethanofurantetrahydromethanopterin formyl-transferase, FwdA-F/FmdA-F formylmethanofuran dehydrogenase, FpoA-O F_420_H_2_ dehydrogenase, HdrDE membrane-bound heterodisulfide reductase, RnfA-G Na^+^-translocating ferredoxin, NAD^+^ oxidoreductase complex, AtpVA-Vk: V/A-type H^+^/Na^+^-transporting ATPase, DMS dimethylsulfide, MeSH methanethiol, CO(III) cobalamin binding protein, CoM coenzyme M, CoB coenzyme B, H_4_MPT tetrahydromethanopterin, MFR methanofuran, F_420_H_2_ reduced coenzyme F_420_, Fd ferredoxin [76].

In addition, three *Methanolobus* MAGs (bin95, bin151, bin173) were assembled from the incubation metagenomes. By searching for genes encoding for proposed enzymes involved in DMS-dependent methanogenesis [76], we were able to reconstruct mostly complete pathways within the three *Methanolobus* MAGs (Fig. 7, Tab. S6). In short: MtsD transfers both methyl groups of DMS to CoM, with MeSH as intermediate and the formation of two CoM-CH_3_. The methyl group can then be transferred to tetrahydromethanopterin (H_4_MPT) via MtrA-H and subsequent CO_2_ production, or it can be transformed into methane under CoB-CoM heterodisulfide (CoB-S-S-CoM) formation. Regeneration of CoM, CoB, coenzyme F_420_, and ferredoxin (Fd_red_) would likely be carried out by the F_420_H_2_ dehydrogenase (FpoA-O), the membrane-bound heterodisulfide reductase (HdrDE), and the Na^+^-translocating ferredoxin complex (RnfA-G), respectively. While a combination of Fpo and HdrD for CoM, CoB and F_420_ regeneration would potentially also be possible [76], other enzymes for Fd_red_ oxidation like the coenzyme F420-reducing hydrogenase (FrhABG), the energy-conserving hydrogenase (EhbA-Q), the [NiFe]-hydrogenase (VhtGACD) were not found in any of the MAGs. Genes encoding for the soluble heterodisulfide reductase HdrABC were present in all MAGs. However, none of the genes for the enzymes that deliver the necessary electrons to this complex could be found in any of the three MAGs (e.g., formate dehydrogenase FdhAB, F420-non-reducing hydrogenase MhvAGD). The H^+^ and Na^+^ membrane potential, generated by their translocation in different steps of the suggested pathway, can be used for ADP phosphorylation by the ATPase (AtpVA-VK).

The specificity of the BLASTp results were confirmed by calculating a phylogenetic tree with the identified MtsDFH sequences (>400 aa length) with a backbone of closely related reference sequences (Fig. S7). The majority of the sequences found in the metagenomes from the incubations were MtsD (36.5 rpkm in DMSO, 48.0 rpkm in DMSO + Mo), followed by MtsH (5.2 rpkm in DMSO, 3.9 rpkm in DMSO + Mo), while MtsF was practically absent (<0.02 rpkm). The presence of *mcrA* and *mtsDH* in *Methanococcoides* and *Methanolobus* indicates their capability for DMS-dependent methanogenesis. In contrast, the *mcrA* gene was not detected in the taxon with the highest *mtsD* counts (*Methanohalophilus*).

## Discussion

Owing to the relevance of organic sulfur compounds for the carbon and sulfur cycle, the individual processes, organisms, and genes involved in aerobic DMS conversion have been studied for decades [77–79]. Most of the cultivation-independent molecular studies have concentrated on DMS and DMSP cycling in the marine realm [15, 80–84], and only a few studies have also considered microbial DMSO reduction [85, 86]. Respiratory DMSO reduction has long been known as a source of DMS, which in turn can be emitted into the atmosphere or microbially degraded to CH_4_ or CO_2_ [20]. The opposing effects of the climate-cooling trace gas DMS and the greenhouse gas CH_4_ require a comprehensive understanding of their environmental fate. While DMSO concentrations have been measured in different marine and limnic waters with the highest values of 620 nmol∙L^−1^ [87–89], to date, there are no measurements from marine sediments available. The highest concentrations measured within our saltmarsh sediments of 194.4 µmol∙L^−1^, calculated by the measured maximum of 106.2 nmol∙g^−1^ and a sediment weight of 1.83 g∙cm^−3^ (Tab. S2), were ~300 times higher. DMSO concentrations added to our incubations (0.1 and 1 mM) may thus not have been unrealistically high. The fast reduction of DMSO observed in incubation experiments (Fig. S4) and DMSO depletion within the first few centimeters of the natural sediments demonstrates the presence of a DMSO-reducing microbial community in saltmarshes. The fate of DMSO and the organisms controlling its turnover in anaerobic marine sediments have remained largely unidentified. Here, we followed up the seminal work by Kiene and Capone [20] using anoxic slurry incubation with DMSO addition, extended by 16S rRNA gene sequencing and shotgun metagenomics. We combined incubation experiments with the analysis of natural saltmarsh sediments to identify the microorganisms driving DMSO reduction and DMS-dependent methanogenesis.

The observed succession of metabolites in our incubation experiments matched patterns found in previous studies [20, 36]. Based on DMS and CH_4_ concentrations in the headspace of the slurry incubations, the process can be separated into two distinct phases: initial DMSO reduction with DMS production (T0 to T1) followed by DMS degradation with methanogenesis (T1 to T2). Although these phases were stimulated by DMSO addition to enrich the organisms driving these processes, they overlapped in the incubations and most likely occur simultaneously in nature or potentially in neighboring regions of the sediment profile in natural settings.

### DMSP, the precursor for organic sulfur cycling

DMSP is considered the main source of DMS in marine environments [90]. Our observations indicate that DMSP and DMSO are readily available in saltmarsh sediments, where a diverse community of heterotrophic bacteria utilizes them. The natural concentrations of DMSP (<300 nmol∙g^−1^) observed on Spiekeroog and in the Stiffkey saltmarsh were within the range of previously reported values [15, 80]. Across the Spiekeroog transect, DMSP concentrations peaked at the shoreline (Edge). The high gene counts of *dsyB* in the lower saltmarsh (Low) hints at an increased contribution of bacterial DMSP production. In contrast, the pioneer zone and saltmarsh edge could be strongly influenced by other highly abundant DMSP producers, such as *Spartina* spp. (synonymous with *Sporolobus* spp.) and diatoms [6, 16, 18].

### Natural DMSO-reducing community readily reacts to DMSO stimulation in batch experiments

DMSO reduction is widespread across bacteria and archaea (e.g., *Actinobacteriota*, *Proteobacteria*, *Halobacterota*, see Tab. S4), likely due to the ancient origin of the DMSO reduction enzyme family [91]. This prevalence was also reflected in the 16S rRNA gene and metagenome analysis of this study’s incubation experiments and natural samples, which reflects a ubiquitous ability for DMSO reduction [92]. Moreover, the organisms and genes linked to DMSO reduction found in the unbinned metagenomes were mostly the same in the natural saltmarshes and the incubation experiments, even though they originated from far distant sites (Stiffkey, UK and Spiekeroog, Germany).

Results of our incubation experiments suggest a negative effect of molybdate on the relative abundance of SRB and most of the other *dmsA*-carrying bacteria. Only a few groups of *dmsA*-carrying bacteria (e.g., *Zoogloeaceae*, *Sporomusaceae*) increased in relative abundance in DMSO+Mo but decreased in DMSO treatments. The *Zoogloeaceae* were the group with the highest *dmsA* gene count in the Spiekeroog metagenomes but have not yet been tested for DMSO reduction in culture. On a lower taxonomic level, the *Zoogloeaceae* and *Sporomusaceae* were assigned to *Thauera* sp. MZ1T and *Acetonema longum*, which are not known for sulfate reduction [93, 94]. Taken together, SRB may not be able to fully compensate for the inhibition of sulfate reduction with DMSO reduction and could be less competitive for DMSO in low-sulfate environments, which supports the findings of Jonkers et al., [95], where *Desulfovibrio desulfuricans*, for example, could not generate growth from DMSO reduction when sulfate reduction was inhibited. In contrast, the Tor-type-carrying bacteria detected in the DMSO + Mo incubations indicate a higher competitiveness of *Rhodobacterales* or *Vibrionales* under low sulfate conditions. In the three *Vibrio* MAGs assembled from the incubation experiment most of the additional genes of the DMSOR gene operons were found in addition to the chosen DMSOR marker genes (*dmsA*, *torA*, *torZ*) [10]. This supports their ability for DMSO reduction, potentially even with more than one pathway.

In previous experiments, TorA was induced by DMSO addition and here it increased in gene counts in our treatments (DMSO, DMSO+Mo), however it was shown that it only has a low affinity to DMSO [96]. Hence, the observed increases in our incubations may also originate from the growth of *Vibrionaceae* and *Shewanellaceae* using one of the other DMSO reduction enzymes (TorZ, DmsA). As of today, there is no dedicated DorA KEGG ortholog, hence we tried to identify DorA homologs with hmm profiles provided within the DiTing-pipeline [86]. This approach reannotated some sequences as DorA which were affiliated with TorA, TorZ, or the biotin sulfoxide reductase BisC based on GhostKOALA and phylogenetic trees (data not shown). The only reviewed DorA sequence on Uniprot (Q52675) is annotated as TorZ by GhostKOALA. Hence, potential DorA sequences were likely included in the TorZ counts with our approach. The high similarity between TorZ, DorA, and BisC poses a challenge to any chosen similarity-based annotation approach. The presence of the twin arginine motif, which is needed for transmembrane transport by the TAT pathway, in the TorZ and TorA sequences and its absence in the BisC sequences further supports the GhostKOALA annotation.

### DMS-dependent methanogenesis and alternative fates of DMS

DMS-dependent methanogenesis requires an initial methyl transfer from DMS to coenzyme M [41]. The two known types of DMS methyltransferases, MtsA and MtsDFH, have been identified in methanogenic cultures [41, 42], but there are only few reports on their occurrence in the environment [97]. These were not found in the investigated saltmarshes, probably due to the low abundance of methanogens in surface sediments, combined with the chosen sequencing depth, poor database representation of *mtsDFH*, and bias from stringent DIAMOND BLASTp search settings. While there might be additional, so far unknown methyltransferases involved, MtsDH seem to be the predominant known DMS methyltransferases. This is indicated by the absence of MtsAF and the enrichment of the MtsDH-carrying *Methanosarcinaceae* and the presence of MtsD in three *Methanolobus* sp. affiliated MAGs in our incubation experiments. While methylotrophic methanogenesis has been observed in *Methanohalophilus* cultures [98], the DMS-degrading members *M. zhilinae* and *M. oregonense* were reclassified to *Methanosalsum zhilinae* and to *Methanolobus oregonensis* [75, 99, 100]. However, the *mcrA* gene is present in all ten publicly available genomes of *Methanohalophilus* (18.04.2023) on IMG (Genome IDs: 2703719298, 2703719067, 2754412526, 2914862632, 2642422606, 2913510308, 646564550, 2806310721, 2914860506, 2706794887), but we could not retrieve any MAGs affiliated to this genus. Based on our findings and the presence of *mcrA* genes in the publicly available genomes, we assume these archaea are also DMS-dependent methanogens, but their *mcrA* has not been detected in the metagenomic analysis. In most of our experiments, the CH_4_ yields were close to the stochiometric maximum of 1.5 for DMS-dependent methanogenesis [74, 101] and accompanied by a strong increase in relative abundance of the methylotrophic *Methanosarcinales*. This supports the findings from Tsola et al., [36], who showed that methanogenesis can be a substantial sink for DMS in anoxic intertidal sediments. The slightly lower CH_4_ yields from DMS, especially when only 0.1 mM DMSO was added, points towards an additional sink for one of the compounds. This supports the hypothesis of higher competitiveness of non-methanogenic DMS degraders, such as SRBs, under low DMS conditions [19, 37, 102]. The higher CH_4_ yield observed in some incubations when sulfate reduction was inhibited offers additional evidence for DMS degradation by SRBs, although it is not entirely conclusive.

The increasing relative abundances of *Thiotrichales* in our anoxic incubation experiments do not contradict their general lifestyle. Primarily, *Thiotrichales* are aerobic sulfur-oxidizing bacteria [103], with some members capable of an anaerobic lifestyle by nitrate reduction [104]. One representative (*Methylophaga thiooxydans*) is capable of aerobic methylotrophic growth on DMS and degradation of sulfide to tetrathionate [105, 106]. Also, they have been observed near *Methanosarcinales* at CH_4_ seeps [103]. Whether and how these organisms may contribute to DMS or sulfide degradation under anoxic conditions must be addressed in future work.

## Conclusions

High DMSO concentrations in saltmarshes highlight its relevance for anaerobic respiration and subsequent DMS production. We found a widespread genetic potential for DMSO reduction in the environment, with a high prevalence of the Dms-type of DMSOR. The relative increase of *mtsDH*-carrying *Methanosarcinacaea* under high DMS concentrations shows the role of DMS as non-competitive substrate for methylotrophic methanogens. However, methanogenesis as DMS-sink might be lower under natural conditions and must be quantified in future investigations. The supposed climate-cooling effect of DMS emissions would be negated by microbial transformation to the greenhouse gases CH_4_ and CO_2_.

### Supplementary information

Supplemental Material

## Data Availability

The raw reads of 16S rRNA amplicon sequencing, the raw reads of the whole genome shotgun sequencing, and nucleotide sequences of MAGs have been deposited in the European Nucleotide Archive (ENA) at EMBL-EBI under accession number PRJEB61311 (https://www.ebi.ac.uk/).
