# Supplementary material for: Microbial drivers of DMSO reduction and DMS-dependent methanogenesis in saltmarsh sediments
Source: ISME J. 2023 Oct 25;17(12):2340–51. doi: 10.1038/s41396-023-01539-1 (PMC10689795; doi:10.1038/s41396-023-01539-1)
Supplement: Supplementary file 1 — Supplemental Material [file 41396_2023_1539_MOESM1_ESM.pdf]

Microbial drivers of DMSO reduction and DMS-dependent methanogenesis in saltmarsh sediments - Supplementary materials

Dennis Alexander Tebbe[1*], Charlotte Gruender[2*], Leon Dlugosch[1], Kertu Lõhmus[3], Sönke Rolfes[1], Martin Könneke[1], Yin Chen[2], Bert Engelen[1] and Hendrik Schäfer[2]

Contact corresponding author: h.schaefer@warwick.ac.uk , +44 24 765 75052

[1]Institute for Chemistry and Biology of the Marine Environment, University of Oldenburg, Carl-von-Ossietzky-Str. 9-11, 26129 Oldenburg, Germany

[2]School of Life Sciences, University of Warwick, CV4 7AL, Coventry, UK

[3]Institute of Biology and Environmental Sciences, University of Oldenburg, Carl-von-Ossietzky-Str. 9-11, 26129 Oldenburg, Germany

* These authors contributed equally to this work

Authors are:

DAT, CG, LD, KL, SR, MK, YC, BE, HS

Contributions:

HS, BE, and DAT conceived the study; DAT, HS, CG, KL, SR, coordinated and carried out fieldwork and acquired data, DAT, GC, LD, YC, HS, BE and MK, analyzed data and interpreted the results; DAT, BE, HS drafted the manuscript, all authors revised the manuscript. The final version was approved by all authors. All authors agreed to be accountable for all aspects of the work in ensuring that questions related to the accuracy or integrity of any part of the work are appropriately investigated and resolved.

Competing Interests: The authors declare no commercial or financial conflict of interest.

Keywords:

Metagenome, 16S rRNA, DMSP, organic sulfur, methylated sulfur, intertidal, microbial communities

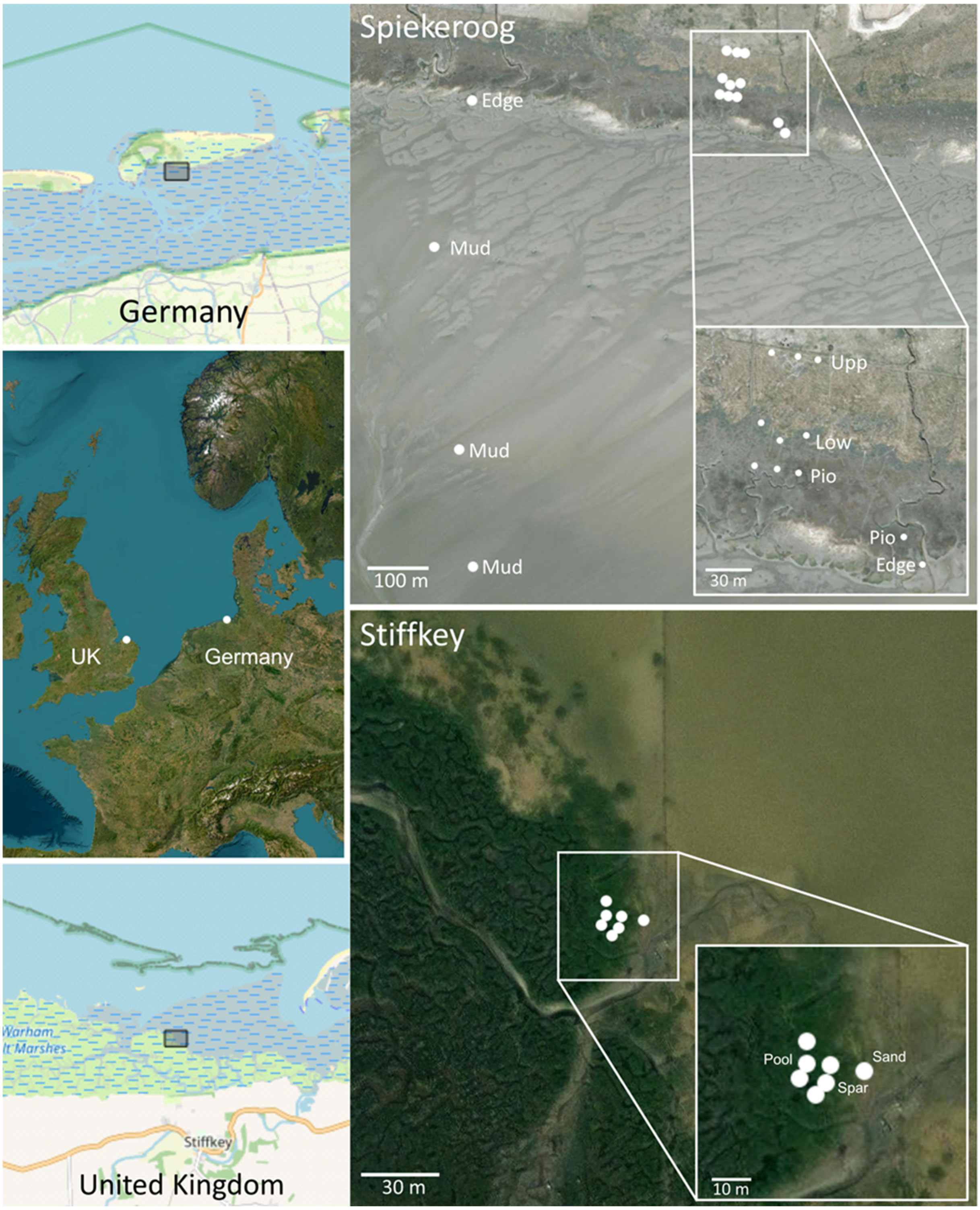

Figure S1: Map of sampling sites on Spiekeroog Island, Germany (top) and Stiffkey salt marsh, UK (bottom). Closeup pictures of representative sites can be found in fig. S2. GPS locations can be found in tab. S1. These maps were made with the leafleat package(1) and World Imagery (2), using the sources: Esri, DigitalGlobe, GeoEye, i-cubed, USDA FSA, USGS, AEX, Getmapping, Aerogrid, IGN,IGP, swisstopo, and the GIS User Community.

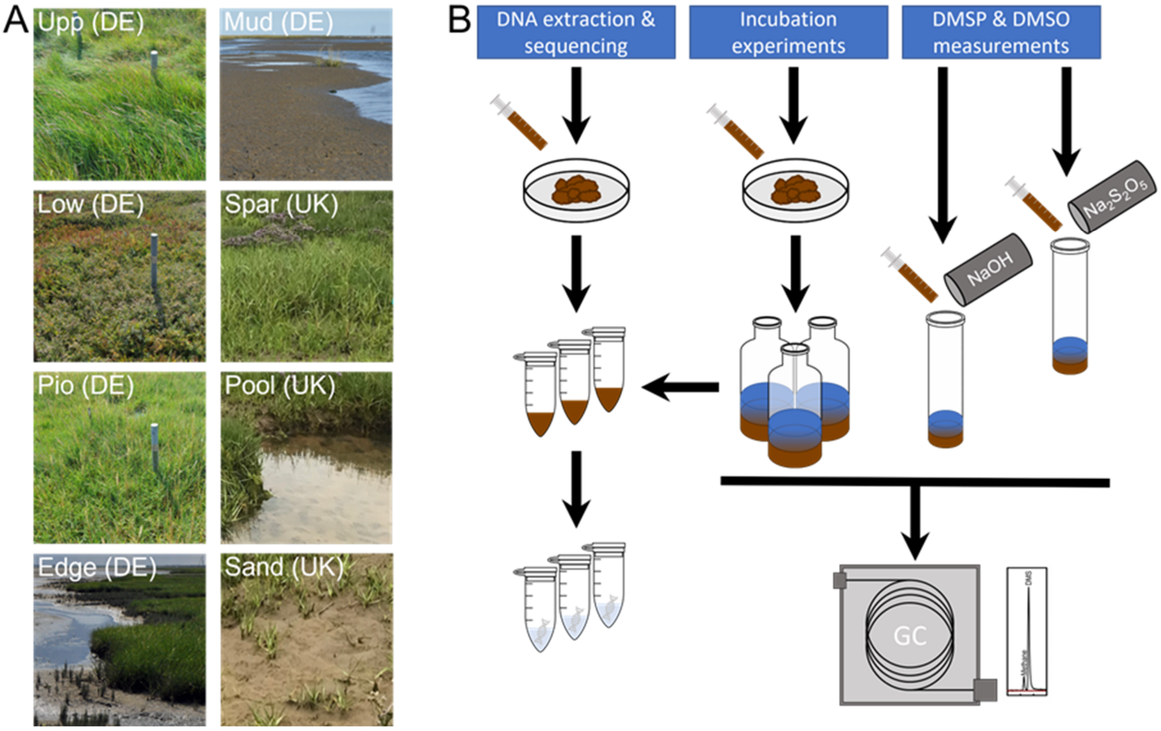

Figure S2: Sampling sites and sample processing. (A) representative pictures of sampling sites on Spiekeroog, Germany (DE) and Stiffkey saltmarsh, United Kingdom (UK). (B) sampling processing scheme. Different amounts, replicates, and depth layers were taken with push cores using cut-off syringes. For molecular analyses and incubation experiments, replicates were mixed to create bulk sediment samples. Technical replicates of chemical profiles were analyzed individually. For incubation experiments, slurries (2cm^3 sed., 20ml sterile anoxic basal medium) were supplemented with 0.1mM DMSO, 1mM DMSO, and 1mM DMSO+Mo, respectively.

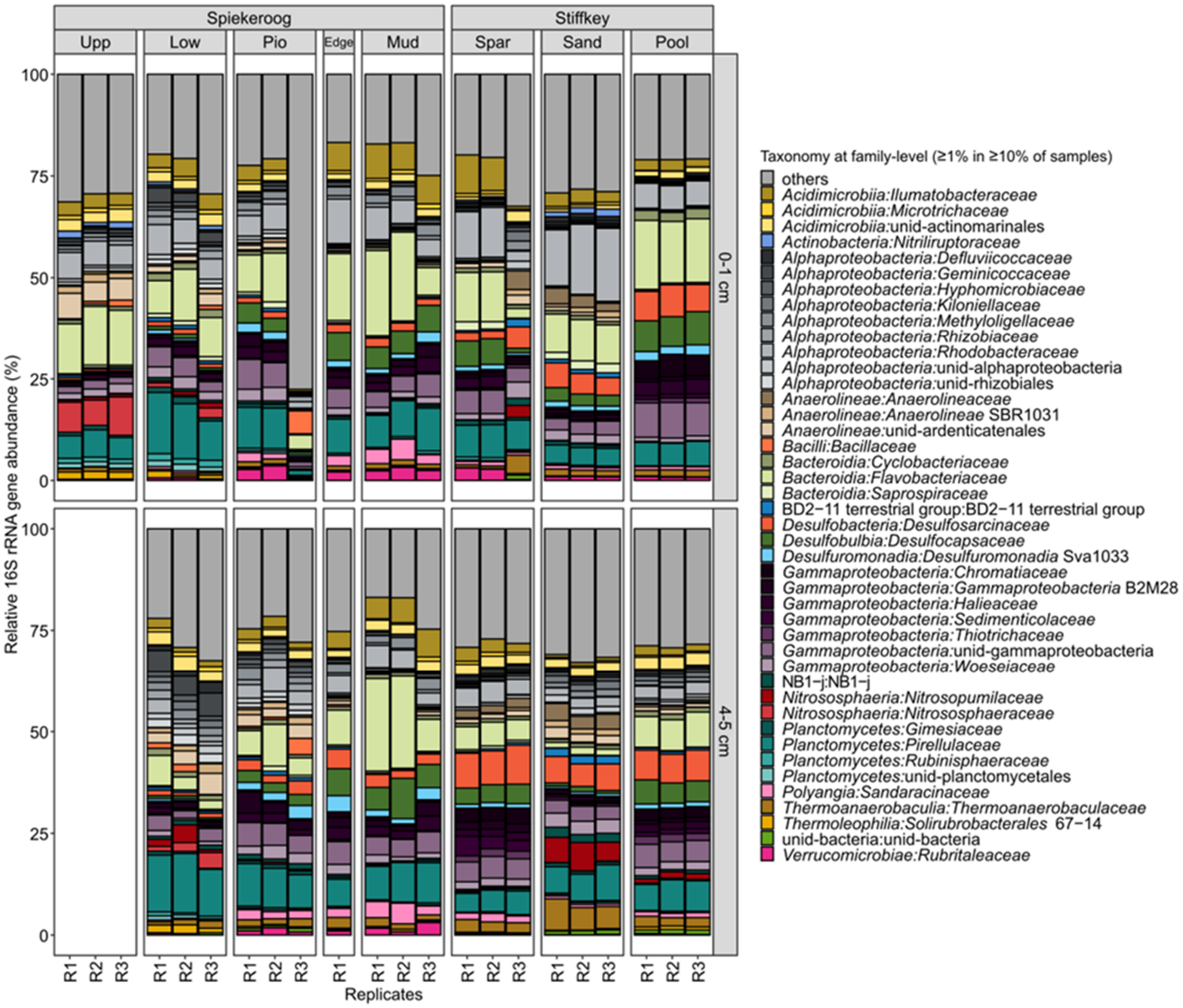

Figure S3: Relative prokaryotic abundances of saltmarsh sediments, based on 16S rRNA gene sequence analysis. Samples from Spiekeroog, Germany (July 2019) and Stiffkey saltmarsh, UK (July 2021) correspond to the respective environmental DMSP and DMSO concentrations (Fig. 2). Sediment for the upper saltmarsh 4-5 cm could not be retrieved. Taxa were grouped at family-level. Taxa with a relative abundance of ≥1 % in ≥10% of samples were displayed, while the remaining were grouped into “others”.

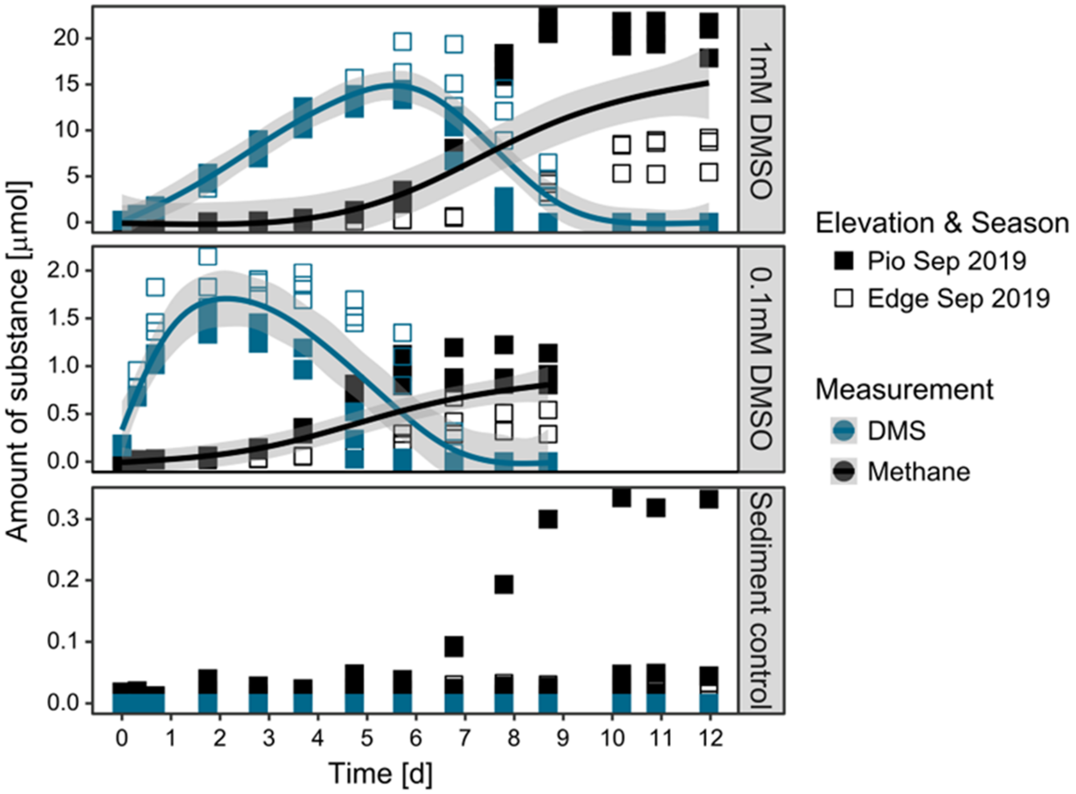

Figure S4: Additional incubation experiments done with sediments taken from Spiekeroog in September 2019.

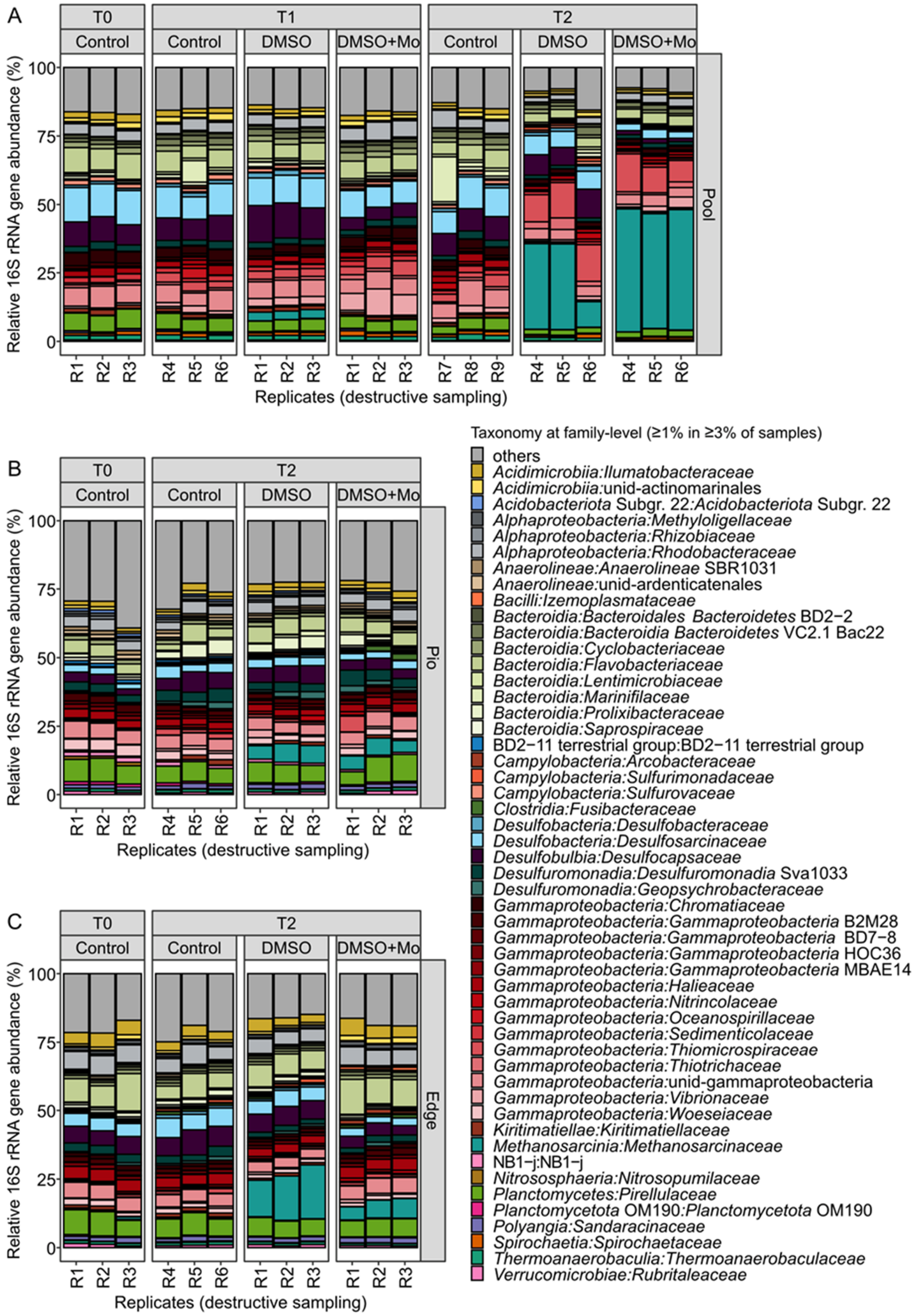

Figure S5: Relative prokaryotic abundances from incubation experiments used for differential gene analysis shown in Fig. 4. Incubations were set up with sediments from A) Stiffkey saltmarsh (Pool), B) the pioneer zone from Spiekeroog (Pio), and C) the edge of Spiekeroog saltmarsh (Edge). Relative abundances are based on 16S rRNA gene sequence analysis. Taxa were grouped at family-level. Taxa with a relative abundance of ≥1 % in ≥3% of samples are displayed, while the remaining were grouped into “others”.

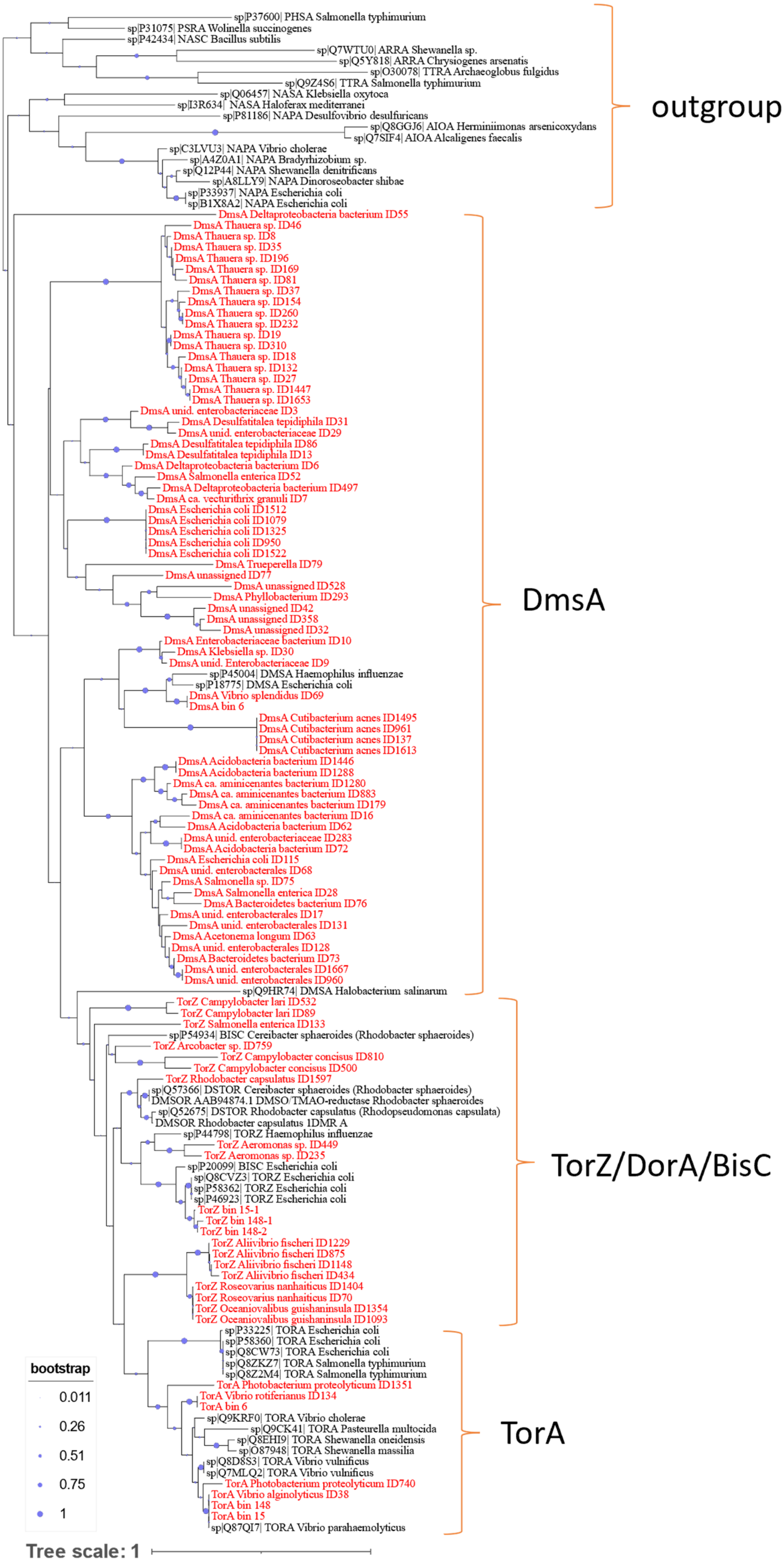

Figure S6: Phylogenetic tree of DMSO reductases. Sequences from this study (red) an "ID" and "bin" number were from the metagenomes and metagenome assembled genomes (MAGs), respectively. Only sequences with a length of >600 amino acids were considered for the alignment. Tree branches can be grouped into the trimethylamine-N-oxide reductase (TorA), and Dimethyl sulfoxide reductase (DmsA). The trimethylamine-N-oxide reductase (TorZ), Dimethyl sulfoxide/trimethylamine N-oxide reductase (DSTOR), and Biotin sulfoxide reductase (BisC) do not from mixed branches, with some taxonomically coherent subbranches. The outgroup contains: thiosulfate reductase molybdopterin-containing subunit (PHSA), polysulfide reductase chain A (PSRA), assimilatory nitrate reductase catalytic subunit (NASC), arsenate respiratory reductase molybdopterin-containing subunit (ARRA), tetrathionate reductase subunit A (TTRA), nitrate reductase (NASA), periplasmic nitrate reductase (NAPA), and arsenite oxidase subunit (AIOA). The evolutionary history was inferred using the Neighbor-Joining method. The percentage of replicate trees in which the associated taxa clustered together in the bootstrap test (1000 replicates) are shown on the branches in blue bubbles. Evolutionary analyses were conducted with an "a la carte" mode (MUSCLE, deletion of gaps columns, JTT distance correction) from phylogeny.fr (3).

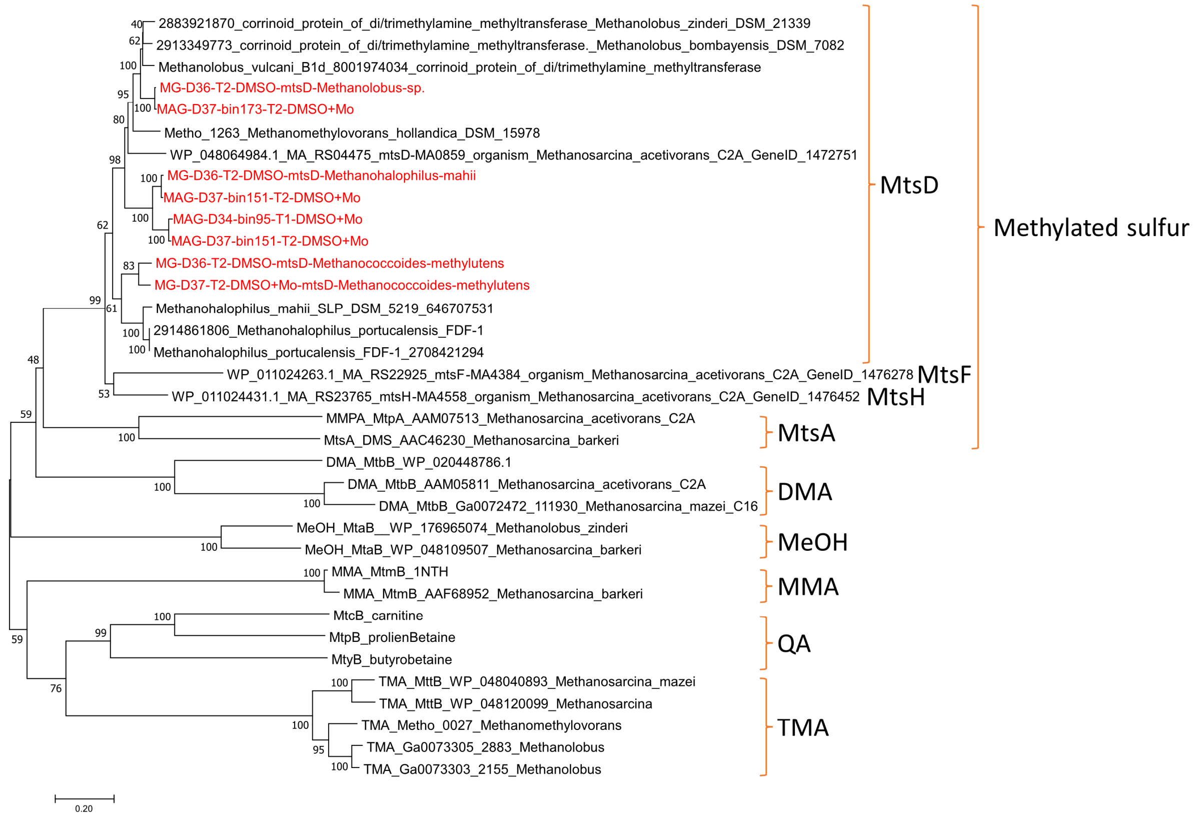

Figure S7: Evolutionary relationships of MT1 methyltransferase domain. Sequences from this study (red) starting with "MG" and "MAG" were from the metagenomes and metagenome assembled genomes (MAGs), respectively. Only sequences with a length of >400 amino acids were considered for the alignment. Tree branches can be grouped into proteins involved in the degradation of methylated sulfur compounds, dimethylamine (DMA), methanol (MeOH), monomethylamine (MMA), quaternary amines (QA), trimethylamine (TMA). The evolutionary history was inferred using the Neighbor-Joining method. The percentage of replicate trees in which the associated taxa clustered together in the bootstrap test (100 replicates) are shown next to the branches. The tree is drawn to scale, with branch lengths in the same units as those of the evolutionary distances used to infer the phylogenetic tree. The evolutionary distances were computed using the Poisson correction method and are in the units of the number of amino acid substitutions per site. The analysis involved 36 amino acid sequences. All ambiguous positions were removed for each sequence pair. There was a total of 731 positions in the final dataset. Evolutionary analyses were conducted in MEGA7 (4).

Table S1: GPS locations of sampling sites.

Salt marsh	Elevation	Purpose	Latitude	Longitude
Spiekeroog	Pio	Environmental data	53.76197567	7.72270620
Spiekeroog	Low	Environmental data	53.76218975	7.72278447
Spiekeroog	Upp	Environmental data	53.76262410	7.72289697
Spiekeroog	Pio	Environmental data	53.76199642	7.72249785
Spiekeroog	Low	Environmental data	53.76216156	7.72252363
Spiekeroog	Upp	Environmental data	53.76264435	7.72270043
Spiekeroog	Pio	Environmental data	53.76201583	7.72227606
Spiekeroog	Low	Environmental data	53.76226340	7.72234301
Spiekeroog	Upp	Environmental data	53.76266697	7.72244678
Spiekeroog	Edge	Environmental data	53.76193020	7.71609429
Spiekeroog	Mud	Environmental data	53.75976583	7.71515670
Spiekeroog	Mud	Environmental data	53.75677067	7.71577220
Spiekeroog	Mud	Environmental data	53.75503080	7.71610861
Spiekeroog	Edge	Incubations, depths profiles	53.76160484	7.72373135
Spiekeroog	Pio	Incubations, depths profiles	53.76144456	7.72391194
Stiffkey	Pool	Incubations	52.96532240	0.92598070
Stiffkey	Pool	Incubations	52.96535900	0.92592140
Stiffkey	Pool	Incubations	52.96538890	0.92594820
Stiffkey	Pool	Incubations	52.96544000	0.92594920
Stiffkey	Pool	Environmental data	52.96538750	0.92603130
Stiffkey	Spartina	Environmental data	52.96535000	0.92601820
Stiffkey	Sand	Environmental data	52.96537310	0.92615470

Table S2: DMSP and DMSO concentrations in natural samples. The mean density is the weight [g] per volume [cm^3] of sample.

Samples			DMSP			DMSO		
Depth (cm)	Date	Site	Mean density ($g \cdot cm^{-3}$)	DMSP ($nmol \cdot g^{-1}$)	No. of replicates	Mean density ($g \cdot cm^{-3}$)	DMSO ($nmol \cdot g^{-1}$)	No. of replicates
0-1	July19	Edge	1.62	299.3	1	NA	NA	NA
0-1	March20	Edge	2.06	40.1	1	2.04	7.1	1
0-1	July19	Low	1.33	149 ± 39.5	3	NA	NA	NA
0-1	March20	Low	1.36	121.8 ± 48.9	3	1.14	17.9 ± 11.4	3
0-1	July19	Mud	2.03	51.3 ± 25.9	3	NA	NA	3
0-1	March20	Mud	2.05	15.3 ± 6.9	3	1.81	4.4 ± 1.9	3
0-1	July19	Pio	1.40	283.8 ± 27.2	3	NA	NA	3
0-1	March20	Pio	1.46	156 ± 50.3	3	1.22	33.3 ± 8.4	3
0-1	July19	Upp	1.10	84.6 ± 5.9	3	NA	NA	3
0-1	March20	Upp	1.29	75.4 ± 26.9	3	1.17	8.5 ± 3.8	3
0-1	Sep19	Edge	1.80	143 ± 3.3	3	1.30	28.1 ± 3.4	3
1-2	Sep19	Edge	1.89	46.6 ± 1.2	3	1.42	7.3 ± 0.5	3
2-3	Sep19	Edge	1.52	30.1 ± 8.6	3	1.35	3.7 ± 0.5	3
3-4	Sep19	Edge	2.02	9.8 ± 0.8	3	1.29	2.1 ± 0.2	3
4-5	Sep19	Edge	1.84	15.8 ± 4.3	3	1.37	1.4 ± 0.3	3
5-6	Sep19	Edge	2.12	14.5 ± 2.4	3	1.32	0 ± 0	3
0-1	Sep19	Pio	1.31	181.5 ± 23.4	3	1.80	49.3 ± 1.9	3
1-2	Sep19	Pio	1.37	62.6 ± 4.8	3	1.87	16.4 ± 4.8	3
2-3	Sep19	Pio	1.32	39.7 ± 2.1	3	1.81	5.9 ± 1.9	3
3-4	Sep19	Pio	1.28	35.4 ± 1	3	1.94	1.6 ± 0.7	3
4-5	Sep19	Pio	1.37	27.2 ± 0.6	3	1.85	1.5 ± 0.1	3
5-6	Sep19	Pio	1.36	26.7 ± 1.1	3	1.83	2.3 ± 0.6	3
0-1	July21	Pool	2.08	4.2 ± 0.1	3	2.01	13.3 ± 2.3	3
1-2	July21	Pool	2.05	1.5 ± 0.1	3	1.96	8.8	1
2-3	July21	Pool	1.91	2.1 ± 0.2	3	1.88	4.2	1
3-4	July21	Pool	1.89	0.2 ± 0	3	1.74	0.8	1
4-5	July21	Pool	1.76	0.2 ± 0	3	1.65	4.1	1
5-6	July21	Pool	1.77	0.3 ± 0	3	1.98	2.9	1
0-1	July21	Sand	1.98	294.6 ± 22	3	1.71	19.8	1
1-2	July21	Sand	1.83	1.4 ± 0.1	3	2.10	0	1
2-3	July21	Sand	1.85	0.2 ± 0	3	1.79	0	1
3-4	July21	Sand	2.02	0 ± 0	3	1.93	0	1
4-5	July21	Sand	1.73	0.1 ± 0	3	1.99	0	1
5-6	July21	Sand	1.89	0.2 ± 0	3	2.15	0.6	1
0-1	July21	Spartina	1.81	150.8 ± 6.4	3	1.83	106.2	1
1-2	July21	Spartina	1.79	0.7 ± 0.1	3	1.89	4.0	1
2-3	July21	Spartina	1.86	0.1 ± 0	3	1.85	3.5	1
3-4	July21	Spartina	1.94	0.1 ± 0	3	1.96	3.4	1
4-5	July21	Spartina	1.78	0.3 ± 0	3	1.95	8.1	1
5-6	July21	Spartina	1.93	0.6 ± 0	3	2.04	4.0	1

Table S3: Maximal mean DMS and CH_4 concentration in incubation experiments, including DMSO to DMS and DMS to CH_4 conversion ratios and the timepoints.

Season	Site	Treatment	DMS max mean	DMS max Time	CH_4 max mean	CH_4 max Time	$\frac{DMS_{(max)}}{DMSO_{(input)}}$	$\frac{CH_{4(max)}}{DMS_{(max)}}$	$\frac{CH_{4(max)}}{DMSO_{(input)}}$
July19	Edge	1mM-DMSO	20.3 ± 2.5	4.0	22.2 ± 0.8	10.0	1.0	1.1	1.1
July19	Edge	1mM-DMSO+Mo	13.2 ± 8.7	5.0	24.3 ± 1.4	11.0	0.7	1.8	1.2
July19	Edge	Sediment control	0.0	NA	0.3 ± 0.3	9.0	inf	inf	inf
July19	Pio	1mM-DMSO	18.8 ± 1.2	6.0	23.6 ± 3.2	10.0	0.9	1.3	1.2
July19	Pio	1mM-DMSO+Mo	21.1 ± 0.5	5.0	26.1 ± 2.4	10.0	1.1	1.2	1.3
July19	Pio	Sediment control	0.0	NA	0.1 ± 0	10.0	inf	inf	inf
July21	Pool	1mM-DMSO	18.4 ± 1.8	5.8	23.8 ± 0.8	11.6	0.9	1.3	1.2
July21	Pool	1mM-DMSO+Mo	14.2 ± 0.6	7.6	21.0 ± 1.8	13.6	0.7	1.5	1.1
July21	Pool	Sediment control	0.0	NA	0.0	NA	inf	inf	inf
Sep19	Edge	0.1mM-DMSO	1.9 ± 0.3	1.8	0.6 ± 0.3	8.7	1.0	0.3	0.3
Sep19	Edge	1mM-DMSO	16.7 ± 2.7	5.7	7.8 ± 2.0	12.0	0.8	0.5	0.4
Sep19	Edge	Sediment control	0.0	NA	0.0	NA	inf	inf	inf
Sep19	Pio	0.1mM-DMSO	1.5 ± 0.1	1.8	1.0 ± 0.2	7.8	0.8	0.7	0.5
Sep19	Pio	1mM-DMSO	13.8 ± 0.5	5.7	21.2 ± 1.0	8.7	0.7	1.5	1.1
Sep19	Pio	Sediment control	0.0	NA	0.1 ± 0.2	12.0	inf	inf	inf

Table S4: Experimentally proven DMSO reducers, with 16S rRNA gene identifier (Entry), Species name (Species), DMSO reduction gene (Gene) and source (publication).

Entry	Species	Gene	Publication
AB480699	*Azospirillum brasilense*	NA	Griebler and Slezak (5)
AB106345	*Bacillus cereus*	NA	Zinder and Brock (6)
AB016721	*Bacillus subtilis*	NA	Zinder and Brock (6)
AB021415	*Brevundimonas diminuta*	NA	Griebler and Slezak (5)
AB021414	*Brevundimonas vesicularis*	NA	Griebler and Slezak (5)
AB021374	*Caballeronia glathei*	NA	Griebler and Slezak (5)
AL111168	*Campylobacter jejuni*	NA	Sellars *et al.,* (7)
AB075768	*Clostridium butyricum*	NA	Zinder and Brock (6)
AB007996	*Comamonas testosteroni*	*dmsA*	Griebler and Slezak (5)
AB020186	*Delftia acidovorans*	NA	Griebler and Slezak (5)
FR733668	*Desulfobacterium niacini*	NA	Jonkers *et al.,* (8)
AF192153	*Desulfovibrio desulfuricans*	NA	Jonkers *et al.,* (8)
U48243	*Desulfovibrio halophilus*	NA	Jonkers *et al.,* (8)
AB252583	*Desulfovibrio vulgaris*	NA	Jonkers *et al.,* (8)
KY499468	*Desulfovibrio halophilus*	NA	Griebler and Slezak (5)
EU700082	*Ectothiorhodospira shaposhnikovii*	NA	Vogt *et al.,* (9)
ARTC01000003	*Enterobacter aerogenes*	NA	Griebler and Slezak (5)
AB244288	*Enterobacter cloacae*	*dmsA*	Griebler and Slezak (5)
AB012212	*Enterococcus faecalis*	NA	Zinder and Brock (6)
CP009789	*Escherichia coli*	*dmsA*	Zinder and Brock (6)
AB377129	*Haemophilus influenzae*	*dmsA*	
CP002922	*Haloarcula hispanica*	*dmsA*	Oren and Trüper (10)
AF034620	*Haloarcula marismortui*	*dmsA*	Oren and Trüper (10)
AB603514	*Halobacterium salinarum*	*dmsA*	Oren and Trüper (10), Muller and DasSarma (11)
CP007551	*Haloferax mediterranei*	*dmsA*	Oren and Trüper (10)
AOHU01000104	*Haloferax volcanii*	*dmsA*	Oren and Trüper (10)
AB004753	*Klebsiella pneumoniae*	NA	Zinder and Brock (6)
EU850806	*Marichromatium gracile*	NA	Vogt *et al.,* (9)
AB023371	*Micrococcus luteus*	NA	Griebler and Slezak (5)
AB540984	*Moorella thermoacetica*	*dmsA*	Rosenbaum *et al.,* (12)
AB098590	*Paracoccus denitrificans*	NA	Griebler and Slezak (5)
AJ291826	*Prosthecochloris aestuarii*	NA	Vogt *et al.,* (9)
AJ291827	*Prosthecochloris vibrioformis*	NA	Vogt *et al.,* (9)
AB079370	*Proteus mirabili*	NA	Griebler and Slezak (5)
AB855738	*Proteus vulgaris*	*dmsA*	Zinder and Brock (6)
JALD01000040	*Providencia alcalifaciens*	*dmsA*	Zinder and Brock (6)
AM040492	*Providencia rettgeri*	*dmsA*	Griebler and Slezak (5)
AB037545	*Pseudomonas aeruginosa*	NA	Zinder and Brock (6)
AB680567	*Pseudomonas alcaligenes*	NA	Griebler and Slezak (5)
AB680166	*Pseudomonas chlororaphis*	NA	Griebler and Slezak (5)
AB021398	*Pseudomonas cichorii*	NA	Griebler and Slezak (5)
AB021396	*Pseudomonas citronellolis*	NA	Griebler and Slezak (5)
AB021401	*Pseudomonas marginalis*	NA	Griebler and Slezak (5)
AF094734	*Pseudomonas mendocina*	NA	Griebler and Slezak (5)
AB008001	*Pseudomonas putida*	NA	Griebler and Slezak (5)
CP001312	*Rhodobacter capsulatus*	*dmsA/dorA*	Kappler and Schäfer (13)
AKBU01000001	*Rhodobacter sphaeroides*	*dmsA/dorA*	Johnson and Rajagopalan (14)
D30778	*Rhodospirillum rubrum*	NA	Griebler and Slezak (5)
AB626116	*Salmonella enterica*	*dmsA*	Zinder and Brock (6)
AB061685	*Serratia marcescens*	*dmsA*	Griebler and Slezak (5)
AJ006084	*Shewanella massilia*	NA	Dos Santos *et al.,* (15)
AE014299	*Shewanella oneidensis*	*dmsA*	Shin *et al.,* (16)
AJ551090	*Shewanella piezotolerans*	NA	Xiong *et al.,* (17)
AB305019	*Staphylococcus aureus*	NA	Zinder and Brock (6)
AF112999	*Thiocapsa roseopersicina*	NA	Vogt *et al.,* (9)
FN293055	*Thiococcus pfennigii*	NA	Vogt *et al.,* (9)
Y12372	*Thiocystis minor*	NA	Vogt *et al.,* (9)
AB021423	*Trinickia caryophylli*	NA	Griebler and Slezak (5)
AF273252	*Wolinella succinogenes*	*dmsA*	Lorenzen *et al.,* (18)

Table S5: Metagenome assembled genomes (MAGs) with genes for DMSO cycling or DMSP production. In total 80 and 331 MAGs with >80% completeness (Comp.) and <5% contamination (Cont.) were assembled from Spiekeroog saltmarsh and Stiffkey incubation metagenomes, respectively. 6 Spiekeroog saltmarsh and 8 Stiffkey incubation MAGs had at least one of the following enzyme homologs: DmsB (K07307), DmsA (K07306), DmsD (K23349), TorA (K07811), TorC (K03532), TorZ (K07812), TorY (K07821), TorS (K07647), TorT (K11930), TorR (K07772), TorD (K03533), DsyB (K24666), and Tmm (K18277).

ENA sample	Sample	Bin	Taxonomy	Length (kb)	Coverage	Comp.	Cont.	DmsB	DmsA	DmsD	TorA	TorC	TorZ	TorY	TorS	TorT	TorD	TorR	DsyB	Tmm
ERS15424378	Mud-Rep1	bin103	*Anderseniella* sp.	4271	7.4	96.6	4.3	-	-	-	-	-	-	-	-	-	-	-	-	1
ERS15424379	Mud-Rep2	bin80	*Anderseniella* sp.	4335	8.0	82.8	1.7	-	-	-	-	-	-	-	-	-	-	-	-	1
ERS15424396	T2-DMSO+Mo	bin24	*Gammaproteobacteria*-GCA-001735895	3415	5.6	84.3	3.4	-	-	-	-	-	-	-	-	-	-	-	-	1
ERS15424380	Mud-Rep3	bin74	unid-ilumatobacteraceae	3762	11.1	94.8	0.9	-	-	-	-	-	-	-	-	-	-	-	1	-
ERS15424384	Low-Rep1	bin28	*Ilumatobacter* sp.	3429	13.9	87.9	0.0	-	-	-	-	-	-	-	-	-	-	-	1	-
ERS15424382	Pio-Rep2	bin55	*Ilumatobacteraceae*-JABSQH01	3359	5.1	87.1	1.9	-	-	-	-	-	-	-	-	-	-	-	1	-
ERS15424395	T2-DMSO	bin76	*Marinobacterium* sp.	3694	13.6	100.0	1.5	-	-	-	-	-	-	-	-	-	-	-	-	2
ERS15424396	T2-DMSO+Mo	bin29	*Marinobacterium* sp.	3571	9.8	95.5	2.6	-	-	-	-	-	-	-	-	-	-	-	-	1
ERS15424393	T1-DMSO+Mo	bin201	*Thiomicrorhabdus* sp.	2324	8.1	82.6	2.1	-	-	-	-	-	-	-	-	-	-	-	-	1
ERS15424395	T2-DMSO	bin27	*Thiomicrorhabdus* sp.	2654	23.5	99.8	1.2	-	-	-	-	-	-	-	-	-	-	-	-	1
ERS15424396	T2-DMSO+Mo	bin145	*Thiomicrorhabdus* sp.	2478	37.8	90.6	3.5	-	-	-	-	-	-	-	-	-	-	-	-	1
ERS15424393	T1-DMSO+Mo	bin15	*Vibrio diabolicus*	4613	17.8	93.7	1.4	-	-	-	1	1	1	1	1	1	1	1	-	-
ERS15424393	T1-DMSO+Mo	bin6	*Vibrio kanaloae*	3586	26.2	88.7	2.6	1	1	1	1	1	-	-	1	1	1	1	-	-
ERS15424394	T2-Cont	bin148	*Vibrio diabolicus*	4420	7.0	91.2	0.8	-	-	-	1	1	2	1	1	1	1	1	-	-

Table S6: Metagenome assembled genomes (MAGs) with genes methanogenesis (mcrA) and DMS-methyltransferases. In total 80 and 331 MAGs with >80% completeness (Comp.) and <5% contamination (Cont) were assembled from Spiekeroog saltmarsh and Stiffkey incubation metagenomes, respectively. Three Stiffkey incubation MAGs had at least one of the following enzyme homologs: McrA (K00399, K00400), MtsA (K16954), MtsB (K16955), MtsD (BLASTp), MtsF (BLASTp), or MtsH (BLASTp). These genes could not be found in any MAG from Spiekeroog saltmarsh.

ENA sample	Sample	Bin	Taxonomy	Length (kb)	Coverage	Comp.	Cont.	McrA	MtsA	MtsB	MtsD	MtsF	MtsH
ERS15424393	T1-DMSO+Mo	bin95	*Methanolobus* sp.	2076	7.5	89.9	0.0	2	-	-	1	-	-
ERS15424396	T2-DMSO+Mo	bin151	*Methanolobus* sp.	2137	554.4	96.0	0.0	2	-	-	2	-	-
ERS15424396	T2-DMSO+Mo	bin173	*Methanolobus* sp.	2212	8.9	88.4	4.7	2	-	-	1	-	-

Literature supplementary materials

1. Graul C. leafletR: Interactive web-maps based on the Leaflet JavaScript library. R package version 0.4-0 ed2016.
2. Esri D, GeoEye, i-cubed, USDA FSA, USGS, AEX, Getmapping, Aerogrid, IGN, IGP, swisstopo, GIS-Anwender-Community. 27.06.2023.
3. Dereeper A, Guignon V, Blanc G, Audic S, Buffet S, Chevenet F, et al. Phylogeny.fr: robust phylogenetic analysis for the non-specialist. Nucleic Acids Res. 2008;36(Web Server issue):W465-9.
4. Kumar S, Stecher G, Tamura K. MEGA7: Molecular evolutionary genetics analysis version 7.0 for bigger datasets. Mol Biol Evol. 2016;33(7):1870-4.
5. Griebler C, Slezak D. Microbial DMSO reduction is widespread among microorganisms and is therefore proposed as a reliable activity parameter. SIL Proceedings, 1922-2010. 2000;27(4):2492-7.
6. Zinder SH, Brock TD. Dimethyl sulphoxide reduction by microorganisms. J Gen Microbiol. 1978;105(2):335-42.
7. Sellars MJ, Hall SJ, Kelly DJ. Growth of *Campylobacter jejuni* supported by respiration of fumarate, nitrate, nitrite, trimethylamine-N-oxide, or dimethyl sulfoxide requires oxygen. J Bacteriol. 2002;184(15):4187-96.
8. Jonkers HM, der Maarel MJEC, Gemerden H, Hansen TA. Dimethylsulfoxide reduction by marine sulfate-reducing bacteria. FEMS Microbiol Lett. 1996;136(3):283-7.
9. Vogt C, Rabenstein A, Rethmeier J, Fischer U. Dimethyl sulphoxide reduction with reduced sulphur compounds as electron donors by anoxygenic phototrophic bacteria. Microbiology (Reading). 1997;143(3):767-73.
10. Oren A, Trüper HG. Anaerobic growth of halophilic archaeobacteria by reduction of dimethysulfoxide and trimethylamineN-oxide. FEMS Microbiol Lett. 1990;70(1):33-6.
11. Muller JA, DasSarma S. Genomic analysis of anaerobic respiration in the archaeon *Halobacterium* sp. strain NRC-1: dimethyl sulfoxide and trimethylamine N-oxide as terminal electron acceptors. J Bacteriol. 2005;187(5):1659-67.
12. Rosenbaum FP, Poehlein A, Daniel R, Muller V. Energy-conserving dimethyl sulfoxide reduction in the acetogenic bacterium *Moorella thermoacetica*. Environ Microbiol. 2022;24(4):2000-12.
13. Kappler U, Schäfer H. Transformations of dimethylsulfide. Met Ions Life Sci. 2014;14:279-313.
14. Johnson KE, Rajagopalan KV. An active site tyrosine influences the ability of the dimethyl sulfoxide reductase family of molybdopterin enzymes to reduce S-oxides. J Biol Chem. 2001;276(16):13178-85.
15. Dos Santos JP, Iobbi-Nivol C, Couillault C, Giordano G, Mejean V. Molecular analysis of the trimethylamine N-oxide (TMAO) reductase respiratory system from a *Shewanella* species. J Mol Biol. 1998;284(2):421-33.
16. Shin HD, Toporek Y, Mok JK, Maekawa R, Lee BD, Howard MH, et al. Iodate reduction by *Shewanella oneidensis* requires genes encoding an extracellular dimethylsulfoxide reductase. Front Microbiol. 2022;13:852942.
17. Xiong L, Jian H, Zhang Y, Xiao X. The two sets of DMSO respiratory systems of *Shewanella piezotolerans* WP3 are involved in deep sea environmental adaptation. Front Microbiol. 2016;7:1418.
18. Lorenzen J, Steinwachs S, Unden G. DMSO respiration by the anaerobic rumen bacterium *Wolinella succinogenes*. Arch Microbiol. 1994;162(4):277-81.
